# Resistance to pirimiphos-methyl in West African *Anopheles* is spreading via duplication and introgression of the *Ace1* locus

**DOI:** 10.1371/journal.pgen.1009253

**Published:** 2021-01-21

**Authors:** Xavier Grau-Bové, Eric Lucas, Dimitra Pipini, Emily Rippon, Arjèn E. van ‘t Hof, Edi Constant, Samuel Dadzie, Alexander Egyir-Yawson, John Essandoh, Joseph Chabi, Luc Djogbénou, Nicholas J. Harding, Alistair Miles, Dominic Kwiatkowski, Martin J. Donnelly, David Weetman, The Anopheles gambiae 1000 Genomes Consortium

**Affiliations:** 1 Department of Vector Biology, Liverpool School of Tropical Medicine, Liverpool, United Kingdom; 2 Centre Suisse de Recherches Scientifiques en Côte d’Ivoire, Abidjan, Côte d’Ivoire; 3 Department of Parasitology, Noguchi Memorial Institute for Medical Research, University of Ghana, Accra, Ghana; 4 Department of Biomedical Sciences, University of Cape Coast, Cape Coast, Ghana; 5 Institut Régional de Santé Publique, Université d’Abomey-Calavi, Benin; 6 Big Data Institute, Li Ka Shing Centre for Health Information and Discovery, University of Oxford, Oxford, United Kingdom; 7 Wellcome Sanger Institute, Hinxton, United Kingdom; University of Exeter, UNITED KINGDOM

## Abstract

Vector population control using insecticides is a key element of current strategies to prevent malaria transmission in Africa. The introduction of effective insecticides, such as the organophosphate pirimiphos-methyl, is essential to overcome the recurrent emergence of resistance driven by the highly diverse *Anopheles* genomes. Here, we use a population genomic approach to investigate the basis of pirimiphos-methyl resistance in the major malaria vectors *Anopheles gambiae* and *A*. *coluzzii*. A combination of copy number variation and a single non-synonymous substitution in the acetylcholinesterase gene, *Ace1*, provides the key resistance diagnostic in an *A*. *coluzzii* population from Côte d’Ivoire that we used for sequence-based association mapping, with replication in other West African populations. The *Ace1* substitution and duplications occur on a unique resistance haplotype that evolved in *A*. *gambiae* and introgressed into *A*. *coluzzii*, and is now common in West Africa primarily due to selection imposed by other organophosphate or carbamate insecticides. Our findings highlight the predictive value of this complex resistance haplotype for phenotypic resistance and clarify its evolutionary history, providing tools to for molecular surveillance of the current and future effectiveness of pirimiphos-methyl based interventions.

## Author summary

Control of mosquito populations via insecticidal tools or interventions is a mainstay of campaigns to reduce malaria transmission. However, especially in sub-Saharan Africa, continued insecticidal selection pressure on the most important species of *Anopheles* malaria mosquitoes has favoured the evolutionary selection of increasingly effective resistance mechanisms. We investigate the genetic basis of resistance to the organophosphate pirimiphos-methyl, the dominant insecticide now used for indoor residual spraying campaigns in Africa. Genome-wide association analysis of a population from Cote d’Ivoire showed that resistant specimens share a unique combination of mutations in one gene, the acetylcholinesterase enzyme, which constitute the prime cause of pirimiphos-methyl resistance. Further testing of these mutations in diagnostic assays involving two major malaria vectors, *A*. *coluzzii* and *A*. *gambiae*, validate their use as informative predictors of pirimiphos-methyl resistance. Using data from a large collection of whole genome sequenced specimens from a broader range of locations (Burkina-Faso, Côte d’Ivoire, Ghana, and Guinea), our evolutionary analyses demonstrate that these mutations emerged in *A*. *gambiae* and transferred into *A*. *coluzzii* by inter-specific hybridisation. Our results show how resistance mechanisms in key malaria vectors have developed and spread, and provide validated tools for molecular surveillance to inform public health campaigns.

## Introduction

Pirimiphos-methyl is an organophosphate insecticide that is widely used in control interventions against populations of the malaria vector *Anopheles*, especially in Africa [1,2]. Since 2013, the World Health Organization (WHO) has recommended the use of pirimiphos-methyl for indoor residual spraying (IRS) interventions, the major anti-vector strategy in malaria control after treated bednet distribution [1,3]. Strategic approaches to vector control often rely on the use of multiple insecticides to avoid or overcome the recurrent emergence of resistance in natural populations [4]. In that regard, various insecticide classes have been used in IRS, with pyrethroids—which target the voltage-gated sodium channel—being the dominant choice until recently [5,6]. However, the increase in pyrethroid resistance in *Anopheles* populations [7] has led to a progressive replacement with acetylcholinesterase-targeting insecticide classes, first the carbamate bendiocarb, and, latterly, the organophosphate pirimiphos-methyl [1]. Pirimiphos-methyl is the active ingredient in the most widely used insecticide for IRS in Africa, the spray formulation Actellic, which is highly effective and has strong residual performance [2,5,8,9]. However, resistance has recently been reported in several populations of African *Anopheles* s.l. [10,11], and though control failures have yet to be reported, it represents a clear threat to the efficacy of IRS strategies.

Mechanisms of resistance to pirimiphos-methyl are poorly understood, but organophosphates, as well as carbamates, all block the action of the acetylcholinesterase enzyme (ACE1, encoded by the *Ace1* gene in mosquitoes) via competitive binding to its active site [12]. Studies in culicine and anopheline mosquitoes have found that non-synonymous mutations in *Ace1* can result in resistance to carbamates and organophosphates other than pirimiphos-methyl [13–15]. The most common mutation in *Anopheles* is a glycine to serine mutation in codon 280 (*G280S*, also known as *G119S* after the codon numbering of a partial crystal structure from the electric ray *Torpedo californica* [13,15,16]), which is located near the active site gorge of ACE1 [17] and decreases its sensitivity to organophosphates and carbamates. This mutation results in reduced sensitivity to the acetylcholine neurotransmitter [18], thus carrying potential fitness costs, as demonstrated in *Culex pipiens* [19]. The resistance allele *280S* has been regularly found in natural *Anopheles* populations: *A*. *gambiae s*.*s*. (henceforth, *A*. *gambiae*) and *A*. *coluzzii* from tropical West and Central Africa [13,20–25]; as well as in *A*. *sinensis* [26]. In addition, *Ace1* is subject to duplication polymorphisms (copy number variants, or CNVs) that co-segregate with the *280S* allele in both *A*. *gambiae* and *A*. *coluzzii*, and enhance resistance to carbamates and organophosphates, such as fenitrothion and chlorpyrifos-methyl [22,24,27–31].

In this study we provide an in-depth investigation of the relationship between *Ace1* mutations and pirimiphos-methyl resistance in *A*. *gambiae* and *A*. *coluzzii* using whole-genome sequenced samples from the *Anopheles gambiae* 1000 Genomes project [32,33], and a wider testing of phenotyped specimens from across West Africa. In addition, we perform a first agnostic genome-wide scan for candidate regions contributing to pirimiphos-methyl resistance in a population of *A*. *coluzzii* from Côte d’Ivoire. Finally, we study the contemporary evolution of *Ace1* to answer unresolved questions on the selective pressures and the pattern of inter-specific introgression associated with the spread of this resistance mechanism. Earlier studies provided support for the idea that a common resistance haplotype under positive selection might have introgressed between species [22,24,27], but they focused on a partial region of the *Ace1* gene and did not address the relationship between introgression and the duplication, which extends beyond the gene [30,34]. Here we leverage population genomic resources from the *Anopheles gambiae* 1000 Genomes to overcome these limitations. Overall, our results demonstrate a widespread and dominant role for *Ace1* mutations in pirimiphos-methyl resistance, provide critical insights into resistance diagnosis, and demonstrate that *280S* alleles and *Ace1* duplications co-occur on a single, swept, resistance haplotype that originated in West African *A*. *gambiae* and later introgressed into *A*. *coluzzii*.

## Results

### Conservation and distribution of *Ace1* resistance mutations in *Anopheles*

We examined the frequency and distribution of the two *Ace1* mutations that have been associated with organophosphate and carbamate resistance in *A*. *gambiae* and *A*. *coluzzii*: the *G280S* non-synonymous single nucleotide polymorphism (SNP), and copy number variation (CNV) polymorphisms of *Ace1* and the surrounding genomic region. *G280S* is sometimes known as *G119S* [15] based on its position in the truncated crystal structure of its homolog in the electric ray *Torpedo californica*, where ACE1 protein structure was first elucidated [16]. Due to a culicine-specific N-terminal insertion in ACE1, the exact position of this conserved codon differs among animal orthologs (S1 Data). We provide a list of homologous codon positions for *Ace1* orthologs from selected animal species, including common insect vectors (S2 Data). Henceforth, we will use *A*. *gambiae* s.l.-based codon coordinates and refer to this SNP as *G280S* (wild-type allele, *wt*: *280G*; resistant allele: *280S*; gene accession number: AGAP001356-RA in AgamP4.12).

**Fig 1:**
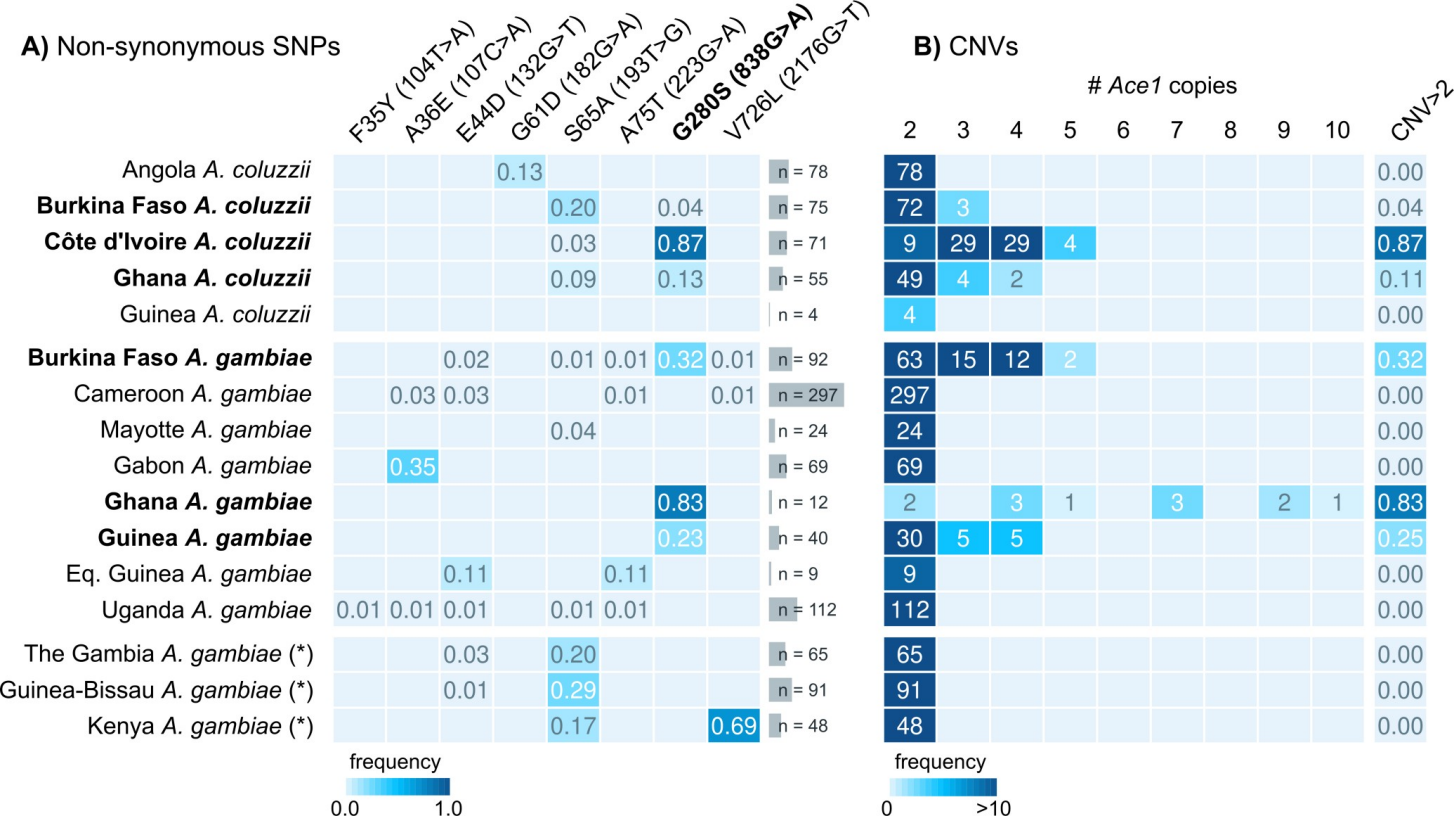
*Ace1* mutations in African populations. **A)** Frequency of non-synonymous SNPs in the *Ace1* gene in African *A*. *gambiae* and *A*. *coluzzii* populations from *Anopheles gambiae* 1000 Genomes, Phase 2. For each SNP, we indicate peptide- and transcript-level coordinates and substitutions. **B)** *Ace1* CNVs across African populations, including the frequency of specimens with >2 copies in each population. A diploid genome without duplications would have 2 copies. Populations where *G280S* and duplications are present are highlighted in bold text. Note: populations denoted with an asterisk (The Gambia, Guinea-Bissau and Kenya) have high frequency of hybridisation and/or unclear species identification.

**Fig 2:**
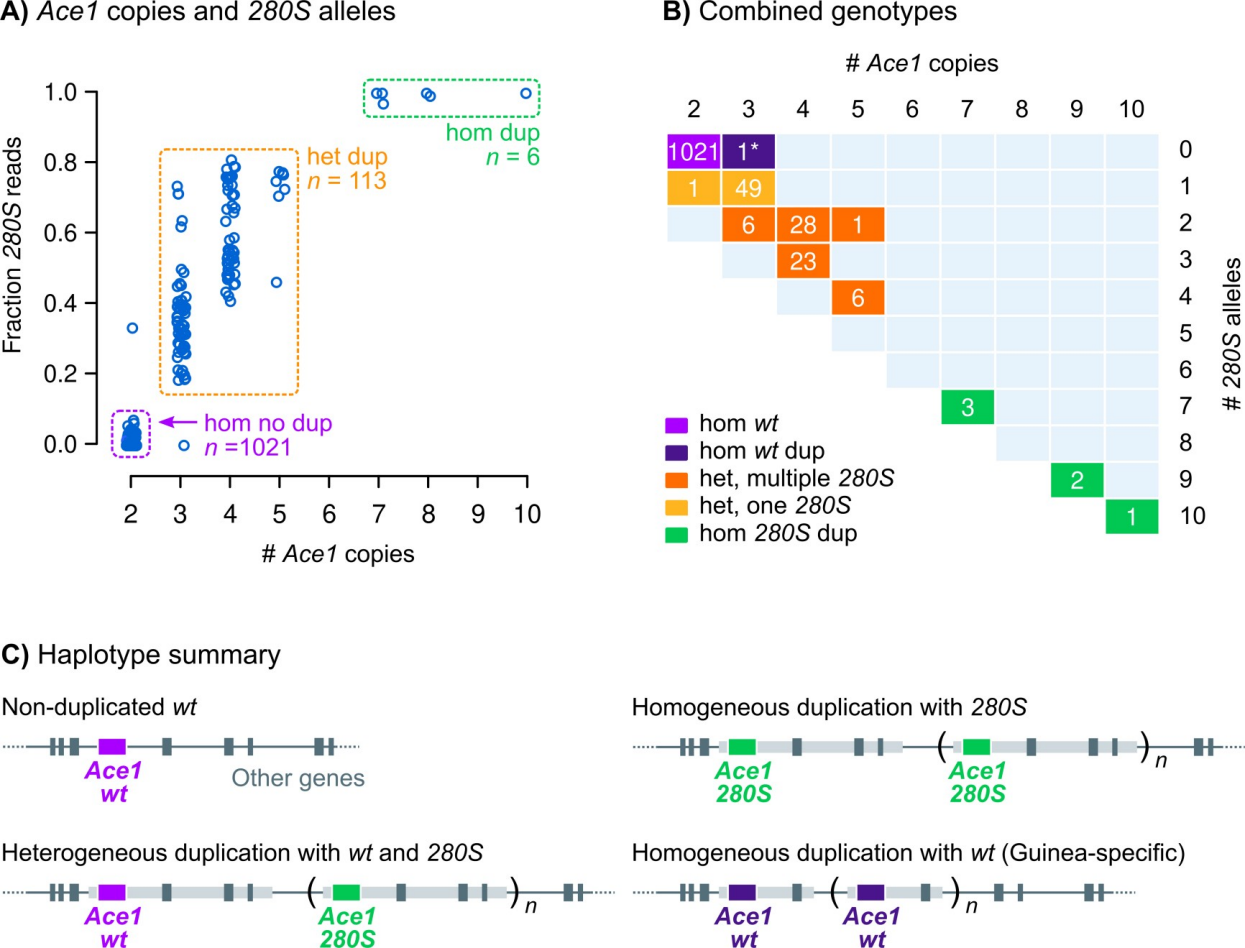
Combinations of *Ace1 G280S* and CNV genotypes. **A)** Fraction of reads supporting *280S* alleles and number of *Ace1* copies (1000 Genomes dataset, *n* = 1142). Boxes highlight groups of haplotypes: non-duplicated *wt* (purple), duplications with both *wt* and *280S* alleles (orange) and duplications with only *280S* alleles (green). **B)** Breakdown of observed genotypes at the *Ace1* locus according to *Ace1* and *280S* copy number. The asterisk (*) denotes a *wt-*homozygous specimen from Guinea that carries an independently evolved *Ace1* duplication. **C)** Diagrammatic summary of *Ace1* haplotypes and their surrounding flanks; light grey rectangles show duplicated regions.

In the *Anopheles* 1000 Genomes cohort (Phase 2, *n* = 1142 genomes; Fig 1) [32], the *280S* resistance allele is present across West African populations of *A*. *coluzzii* (Côte d’Ivoire, Burkina Faso, Ghana) and *A*. *gambiae* (Burkina Faso, Ghana, Guinea), with the highest frequencies observed in Ghanaian *A*. *gambiae* (83% of specimens carry *280S* alleles) and Ivorian *A*. *coluzzii* (87%; Fig 1A), which is consistent with previous results [20–22,27]. According to the database of CNVs in the 1000 Genomes dataset generated by Lucas *et al*. [34] (which we have used in our analyses) *Ace1* duplications have a similar distribution to the SNP in West African populations, as they overwhelmingly overlap with specimens also carrying *280S* alleles (Figs 1B and 2; see below).

The combination of CNVs and *280S* alleles results in multiple genotypes being observed in the *Ace1* locus, defined by a variable number of *280S* copies. We used the fraction of sequencing reads supporting the *wt* and *280S* alleles to estimate the number of *280S* alleles in each sample (Fig 2A and 2B), which revealed that most specimens with duplications carry both *wt* and *280S* alleles at various frequencies (*n* = 113, orange box in Fig 2A), whereas the vast majority of non-duplicated specimens only have *wt* alleles (*n* = 1021, purple box in Fig 2A). In addition, we identify six specimens with high *Ace1* copy numbers that only carry *280S* alleles, present in the Ghanaian *A*. *gambiae* population (Fig 1B).

Virtually all *Ace1* CNVs identified in the 1000 Genomes cohort share the same duplication breakpoints [34], spanning a region *ca*. 200 kbp that includes a total of 11 genes (S5 Data). These breakpoints coincide with those of the strict tandem duplications identified by Assogba *et al*. in *A*. *gambiae* and *A*. *coluzzii* samples from the same region [30] (S17 Data). Fig 2C summarises the combinations of *280S* and duplications that can be observed in this dataset: (i) non-duplicated loci with *wt Ace1*; (ii) chromosomes with *Ace1* tandem duplications carrying *wt* and *280S* alleles (heterogeneous duplications); and (iii) chromosomes containing multiple *280S* alleles (homogeneous duplications). In addition to these three model haplotypes, one specimen from Guinea carries a distinct *wt-*homogeneous duplication (asterisk in Fig 2B). This Guinea-specific duplication is shorter than the one found in all other samples and has different breakpoints (*ca*. 70 kbp, including *Ace1* and one other gene; S4 Data), implying an independent origin. Henceforth, all mentions of *Ace1* CNVs will refer to the major duplication. The absence of specimens carrying the major duplication and lacking *280S* alleles strongly implies that this CNV contains both *280S* and *wt* alleles on the same chromosome.

In addition to *G280S*, we found seven non-synonymous SNPs in *Ace1* with at least 1% frequency in at least one population (Fig 1A). None were in linkage disequilibrium with *G280S* (S3 Data), nor have any of them been previously associated with resistance [15]. The most salient of these additional mutations was a serine-to-alanine SNP in codon 65 (*S65A*), with frequencies of up to 30% in multiple West African populations. Codon 65 maps to the anopheline-specific N-terminal insertion of ACE1 proteins (S1) Data, which lacks predicted secondary structure and is far away from the active-site gorge of the enzyme [17]. This position is not conserved across ACE1 orthologs, being variously encoded as alanine, serine or threonine in different *Anopheles* species (S1 Data).

### Pirimiphos-methyl resistance in Ivorian *A*. *coluzzii* is linked to *Ace1* duplications and multiple *280S* alleles

**Fig 3:**
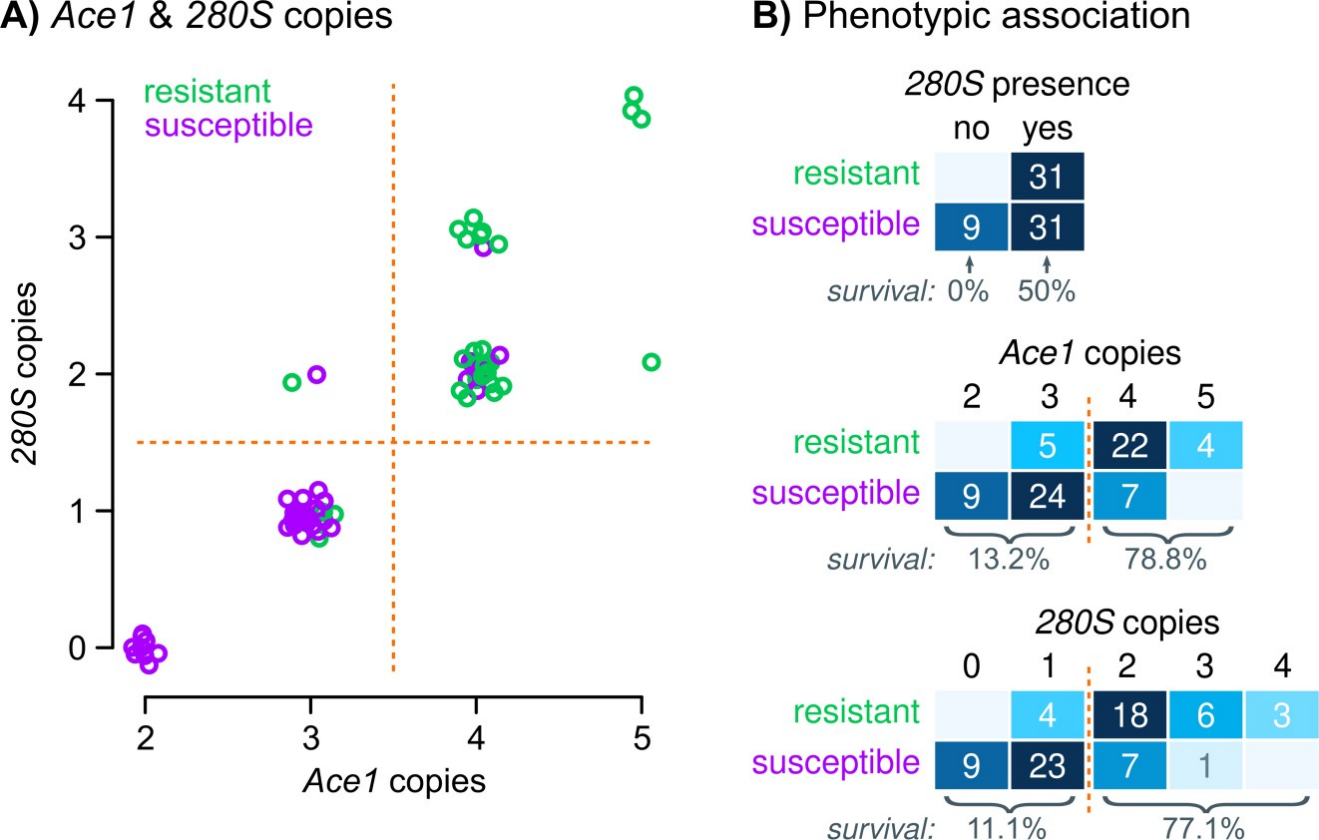
Genotype-phenotype association in Ivorian *A*. *coluzzii*. **A)** Number of *Ace1* copies compared to the estimated number of *280S* copies in Ivorian *A*. *coluzzii* (n = 71), color-coded according to resistance phenotypes. Random jitter has been added for clarity. **B)** Cross-tabulation of pirimiphos-methyl resistance and three *Ace1* mutations in Ivorian *A*. *coluzzii*: *280S* allele presence, number of *Ace1* copies, and number of *280S* alleles. Orange lines denote *ad hoc* groups of genotypes where we identify changes in survival rates (included at the bottom of each table). Source data available in S6.

Next, we examined the association between *Ace1* mutations and resistance to pirimiphos-methyl (Fig 3). We used 71 *A*. *coluzzii* female mosquitoes from Côte d’Ivoire, collected from rice fields in Tiassalé in 2012, that had been tested for resistance prior to genome sequencing. The *G280S* SNP and CNV co-occur at high frequencies in this population, with 87.3% of specimens carrying heterogeneous duplications and one or more *280S* alleles (Fig 3A). Specimens with at least one *280S* allele had a survival rate of 50%, as opposed to 0% in the *wt* homozygotes (Fig 3B), suggesting that *280S* is linked to pirimiphos-methyl resistance in this population (*p =* 3.7 × 10^−3^ in Fisher’s exact test, Woolf-corrected odds ratio = 19.0 [95% confidence interval = 1.1–340.6]) but it does not fully explain the resistance phenotype. The only other non-synonymous SNP present in Ivorian *A*. *coluzzii*, *S65A*, was not associated with pirimiphos-methyl resistance (*p =* 0.605 Fisher’s exact test; S6A and S6B Data).

We found a strong bias towards higher *Ace1* copy numbers in resistant specimens, all of which had at least 3 copies of the *Ace1* locus and most of them had 4 or more (Fig 3B; odds ratio from a binomial generalised linear model [GLM] = 16.9 [5.1–56.6]; *p* = 6.4 × 10^−10^ in a *χ*^*2*^ test compared to a null model). Concordantly, the estimated number of *280S* alleles was also associated with resistance (GLM odds ratio = 10.7 [3.6–31.7]; *p* = 8.9 × 10^−10^ in *χ*^*2*^ test): survival rates increased with higher number of *280S* copies (Fig 3B), with a threshold apparent between individuals with zero or one copies of *280S* (11.1% survival) and those with two or more (77% survival). Crucially, the group of duplicated specimens with one *280S* and two *wt* alleles (*n* = 36), did not show a statistically significant increase in survival compared to non-duplicated *wt* specimens (Fisher’s exact test *p =* 0.553; Woolf-corrected odds ratio = 3.6 [0.2–74.3]).

We examined the combined effects of these mutations (*280S* presence, CNVs and number of *280S* alleles) using binomial GLMs and a step-wise variable addition procedure (S6B Data). Starting from a basic model based on the presence of *280S*, we found that adding the number of *280S* copies improved its predictive ability (at *p* = 2.56 × 10^−7^ in a *χ*^*2*^ ANOVA test), and so did the addition of CNVs (at *p* = 1.96 × 10^−7^). On the other hand, a full model with all three mutations was not significantly better than simpler models excluding either of the two duplication-related variables (at *p* = 0.16–0.22 in *χ*^*2*^ tests), and it was also not significantly better than a minimal model with CNVs as the only remaining variable, obtained according to the Bayesian Information Criterion (*p* = 0.44). Together, these results suggest that duplication-related mutations are necessary to explain the observed levels of resistance in this population, although it is difficult to disentangle their effects due to the fact that they co-occur in the same specimens in this dataset.

### Pirimiphos-methyl resistance diagnostics are repeatable across *A*. *gambiae* and *A*. *coluzzii* populations

We investigated the extent to which the role of *Ace1* and *280S* copy numbers in pirimiphos-methyl resistance could be generalised to other populations beyond the *A*. *coluzzii* from Tiassalé. Specifically, we surveyed *Ace1* mutations and phenotypic resistance in nine West African populations of *A*. *gambiae* and *A*. *coluzzii* from additional locations in Côte d’Ivoire, Togo, and Ghana (Table 1 and Table 2; list of specimens in S6C and S6D Data).

**Table 1:** Association of resistance genotypes with pirimiphos-methyl resistance in nine West African populations of *A*. *coluzzii* and *A*. *gambiae*. For each population, we report number of sampled specimens (*n*), the number of specimens with only *wt*, only *280S*, and both alleles; survival frequency among mutated groups, and the odds ratios of survival in specimens with *280S* alleles (if available) in a binomial generalised linear model, and the *p-*value of this model in a *χ*^*2*^ ANOVA comparison with a null model. Odds ratios (OR) are reported with 95% confidence intervals. Dagger signs (†) denote survival rates in genotypes with fewer than 5 specimens. Samples listed in S6C Data; statistical analysis in S6D Data. Country abbreviations: CI, Côte d’Ivoire; GH, Ghana; TG, Togo.

Population	*n*	Frequencies *wt / wt*+*280S / 280S*	% survival *wt*+*280S*	OR *wt*+*280S*	% survival *280S*	OR *280S*	*p*
*coluzzii* Aboisso (CI)	55	42 / 13 / 0	46.1%	35.1 (3.6–338.0)	*-*	*-*	1.3 × 10^−4^
*coluzzii* Korle-Bu (GH)	214	25 / 165 / 24	49.6%	4.2 × 10^7^ (0 –Inf)	95.8%	9.8 × 10^8^ (0 –Inf)	1.2 × 10^−13^
*coluzzii* Madina (GH)	42	2 / 37 / 3	37.0%	0.4 (0.02–6.5)	100% (†)	4.3 × 10^7^ (0 –Inf)	0.023
*coluzzii* Weija (GH)	131	60 / 70 / 1	54.1%	7.3 (3.0–17.6)	100% (†)	3.7 × 10^7^ (0 –Inf)	2.4 × 10^−6^
*gambiae* Aboisso (CI)	82	3 / 45 / 34	77.8%	7.0 (0.6–85.4)	97%	66.0 (2.9–1491.2)	3.7 × 10^−3^
*gambiae* Madina (GH)	172	19 / 112 / 41	58.9%	12.2 (2.7–55.4)	100%	9.8 × 10^8^ (0 –Inf)	4.4 × 10^−14^
*gambiae* Obuasi (GH)	140	0 / 28 / 112	17.8%	*-*	58.9%	6.6 (2.3–18.6)	6.0 × 10^−5^
*gambiae* Weija (GH)	13	7 / 4 / 2	75% (†)	7.3 × 10^17^ (0 –Inf)	100% (†)	2.6 × 10^9^ (0 –Inf)	1.6 × 10^−3^
*gambiae* Baguida (TG)	102	0 / 14 / 88	57.1%	*-*	56.8%	0.9 (0.3–3.1)	0.982

**Table 2:** Association of *Ace1* duplications with pirimiphos-methyl resistance in specimens carrying both *280G* and *280S* alleles, from nine West African populations of *A*. *coluzzii* and *A*. *gambiae*. For each population, we report number of sampled specimens (*n*), and the results of three binomial generalised linear models (GLM): (i) using the number of *Ace1* copies as the predictor variable, (ii) using the *280S*-to-*280G* allelic ratio, and (iii) a minimal model obtained with step-wise reduction, according to the Bayesian Information Criterion (BIC). For each model, we report the *p*-value from a comparison with a null model (ANOVA *χ*^*2*^ test) and odds ratios (OR) with 95% confidence intervals for the variables included in the model. Sample phenotypes and genotypes listed in S6E Data; statistical analysis in S6F Data. Country abbreviations: CI, Côte d’Ivoire; GH, Ghana; TG, Togo. Note that the Weija *A*. *gambiae* population was excluded from this analysis due to small sample size.

Population	*n*	GLM *Ace1* copies (single variable)		GLM *280S/280G* allelic ratio (single variable)		Minimal GLM (BIC)		
		OR	*p*	OR	*p*	OR *Ace1* copies	OR allelic ratio	*p*
*coluzzii* Aboisso (CI)	11	1.3 (0.7–2.3)	0.361	52.9 (0.01 – 2.2 × 10^5^)	1.3 × 10^−3^	*-*	52.9 (0.01 – 2.2 × 10^5^)	1.3 × 10^−3^
*coluzzii* Korle-Bu (GH)	46	1.8 (1.2–2.6)	1.0 × 10^−4^	2.8 (1.6–5.1)	4.1 × 10^−7^	1.5 (0.9–2.3)	2.3 (1.3–4.0)	3.0 × 10^−7^
*coluzzii* Madina (GH)	34	1.4 (1.04–1.8)	0.013	71.5 (3.3–1539.8)	9.9 × 10^−4^	-	71.5 (3.3–1539.8)	9.9 × 10^−4^
*coluzzii* Weija (GH)	21	0.5 (0.3–1.0)	0.013	3.4 (0.4–28.6)	0.021	0.4 (0.1–1.1)	50 (0.3–9505.0)	2.9 × 10^−3^
*gambiae* Aboisso (CI)	31	1.0 (0.6–1.5)	0.933	2.6 (0.3–24.9)	0.382	*-*	*-*	*-*
*gambiae* Madina (GH)	19	5.2 (1.1–23.9)	7.7 × 10^−3^	6.5 × 10^85^ (0 –Inf)	2.1 × 10^−6^	8.7 × 10^−20^ (0 –Inf)	5.5 × 10^45^ (0 –Inf)	2.6 × 10^−9^
*gambiae* Obuasi (GH)	26	1.5 (0.9–2.3)	0.038	3.1 (0.85–10.8)	0.038	1.5 (0.9–2.3)	*-*	0.038
*gambiae* Weija (GH)	0	-	-	-	-	*-*	*-*	*-*
*gambiae* Baguida (TG)	13	1.4 (0.7–2.6)	0.350	2.9 (0.1–69.4)	0.498	*-*	*-*	*-*

Pirimiphos-methyl resistance was associated with *G280S* mutations in seven out of nine populations (*p <* 0.01 in a *χ*^*2*^ test comparing GLM of each genotype to a null model; Table 1). However, whilst *280S* homozygotes were strongly associated with resistance in populations where they were present in significant numbers (e.g. 95.8% survival rate in *A*. *coluzzii* from Korle-Bu), specimens with both *280S* and *wt* alleles often exhibited lower, intermediate survival rates (e.g. 49.6% in *A*. *coluzzii* from Korle-Bu; six out of nine populations had survival rates <60%; Table 1) similar to that of the *A*. *coluzzii* population from Tiassalé (Côte d’Ivoire) mentioned above (Fig 3A).

Following our findings for the Tiassalé population (Fig 3), we further investigated the phenotypic variation in specimens with both *280S* and *wt* alleles using two CNV-related variables: (i) the ratio of *280S* to *280G* alleles (measured as the ratio of FAM-to-HEX dye signal in a TaqMan qPCR assay) [35]; and (ii) the estimated number of *Ace1* copies, normalised to two single-copy references genes, and assessed relative to the *wt* non-duplicated Kisumu *A*. *gambiae* colony [28] (see Methods).

Pirimiphos-methyl resistance was associated with CNV-related measures in specimens carrying both *wt* and *280S* alleles from *A*. *gambiae* and *A*. *coluzzii* populations, according to generalised linear models that included *280S* allelic ratios and/or *Ace1* copy number as potential informative markers (Table 2). In *A*. *coluzzii* from Korle-Bu and Weija, as well as *A*. *gambiae* from Madina, both the *280S* allelic ratio and the *Ace1* copy number were included in the minimal model (at *p <* 0.01 in a *χ*^*2*^ comparison with a null model); whereas in *A*. *coluzzii* from Aboisso and Madina the allelic ratio was the only variable associated with resistance (at *p <* 10^−3^ in *χ*^*2*^ tests). In Obuasi *A*. *gambiae* specimens with both *wt* and *280S* alleles, the minimal model only included *Ace1* copy number (at *p* = 0.038) but neither measure was strongly predictive, as was the case in *A*. *gambiae* from Aboisso and Baguida. Overall, these results indicate that the combination of *280S* allelic ratios and CNVs provides a similar pirimiphos-methyl resistance mechanism in both species. However, diagnostic capacity appears to vary among populations, suggesting the possible existence of additional resistance mechanisms.

### Genome-wide identification of pirimiphos-methyl resistance variants in Ivorian *A*. *coluzzii*

**Fig 4:**
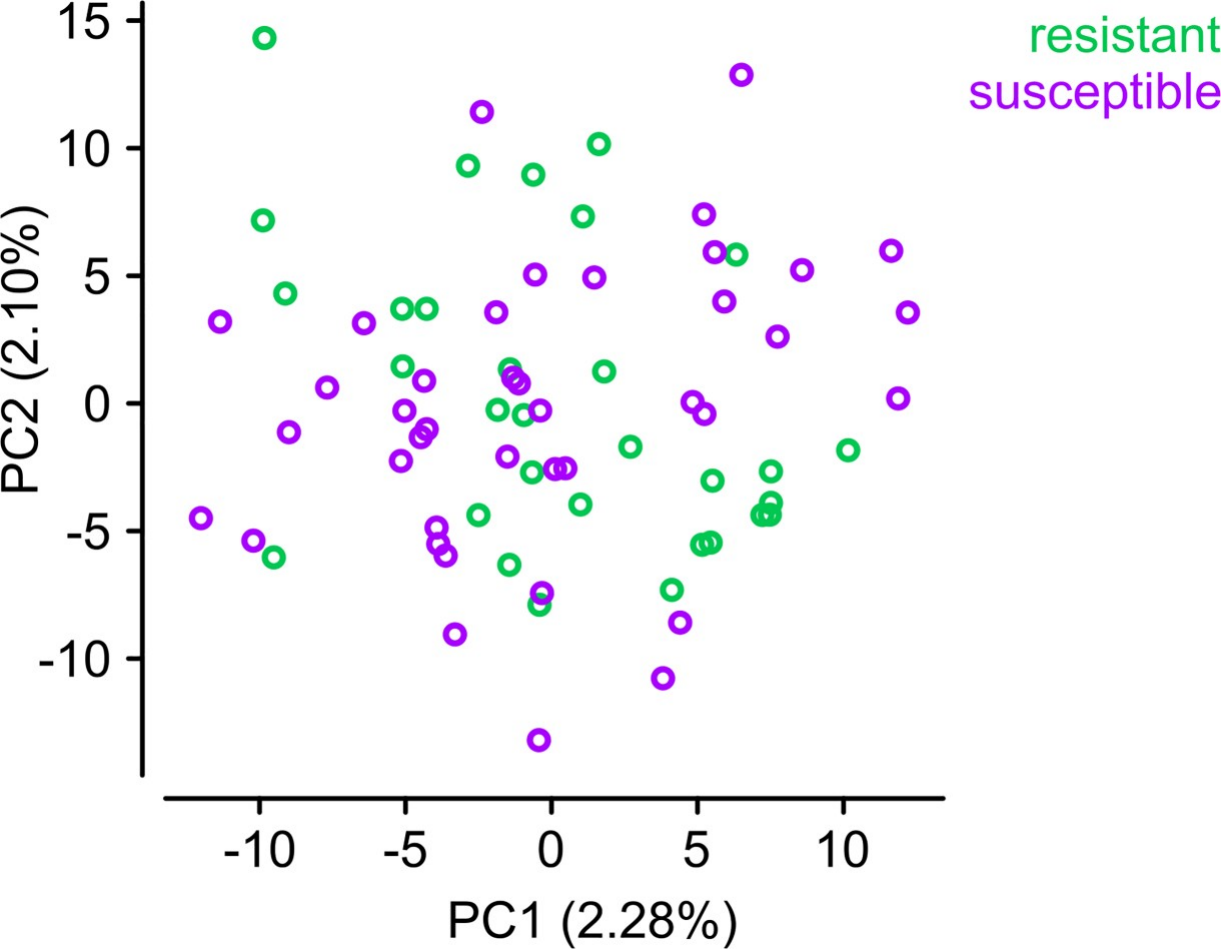
Principal component analysis of Côte d’Ivoire *A*. *coluzzii*. PCA constructed using genotypes of 791 unlinked variants from chromosome 3.

We examined the genomes of the 71 *A*. *coluzzii* specimens from Côte d’Ivoire to identify additional genetic variants—other than *Ace1 G280S* and duplications—that could be linked to pirimiphos-methyl resistance. All Ivorian specimens had been collected in the same location and time (Tiassalé, 2012), before the widespread adoption of pirimiphos-methyl in IRS interventions [1,3]. The absence of a long period of pirimiphos-methyl use prior to collection implies that adaptation to this insecticide would presumably be caused by cross-resistance with previously employed insecticides and/or standing variation, rather than novel selective sweeps. In accordance with this expectation of low differentiation, a principal component analysis of genetically unlinked variants (see Methods) did not reveal population stratification between resistant and susceptible mosquitoes (Fig 4), and average Hudson’s *F*_*ST*_ between them was low along all chromosomal arms (*F*_*ST*_ *≈* 0%).

**Fig 5:**
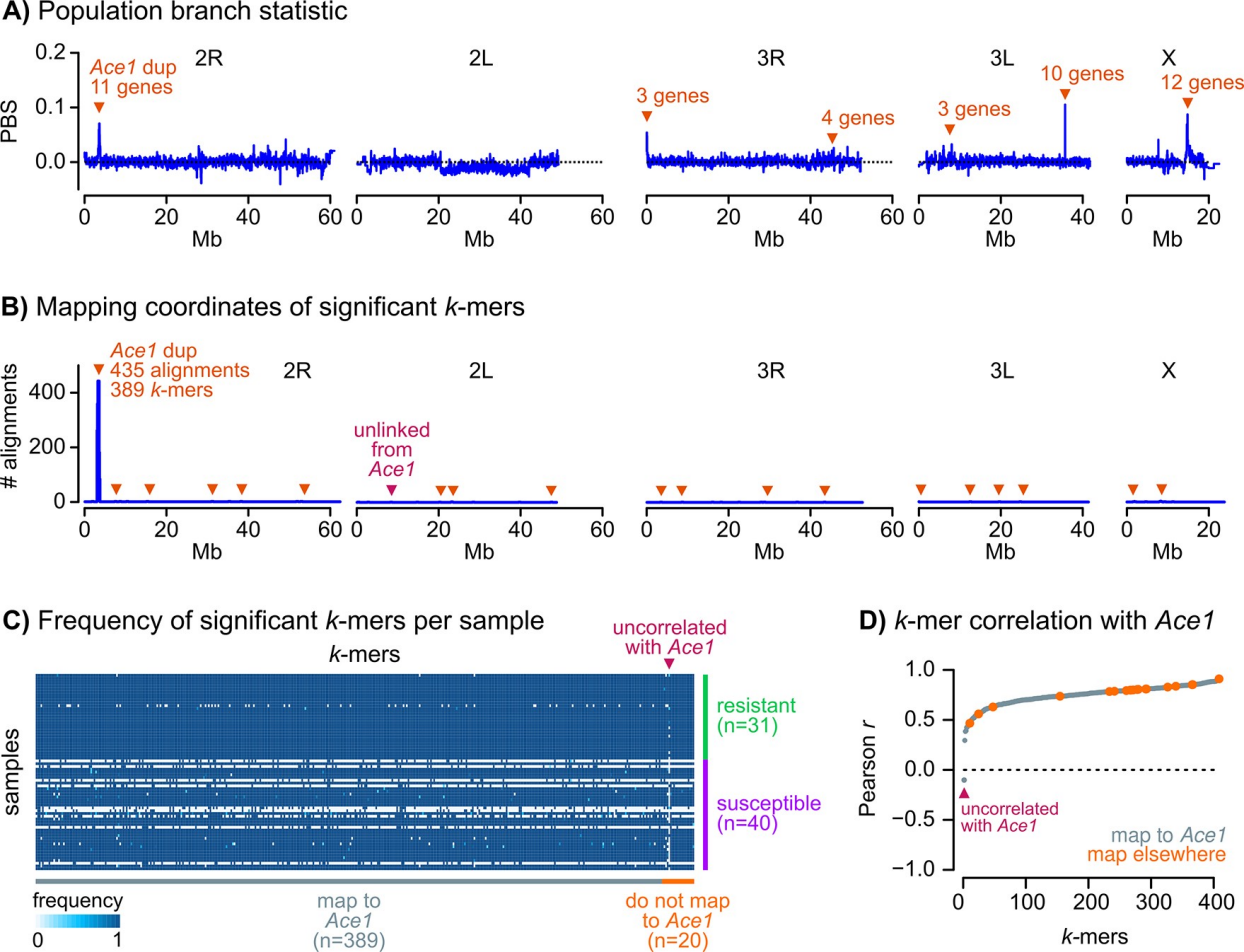
Genome-wide scan of variants associated with pirimiphos-methyl resistance in Ivorian *A*. *coluzzii*. **A)** Profile of population branching statistics along all chromosomal arms, calculated in consecutive blocks of 1000 segregating variants, using resistant and susceptible Ivorian *A*. *coluzzii* as populations A and B, and Angolan *A*. *coluzzii* as outgroup. Orange triangles indicate windows with extreme *PBS* values (*p-*values derived from a standardised distribution of *PBS* along each chromosomal arm, and *FDR* < 0.001), and the number of genes therein. **B)** Mapping coordinates of *k-*mers significantly associated with pirimiphos-methyl (*n* = 439). Most *k*-mers map to the *Ace1* duplication region (*n* = 414) or, despite mapping elsewhere in the genome (*n* = 24), are correlated with *Ace1* copy number (orange triangles). Only one *k-*mer mapping outside of the *Ace1* duplication is not correlated with *Ace1* copy number (purple triangle). **C)** Normalised frequency of each significant *k*-mer (*n* = 439, horizontal axis) in each genome (*n* = 71, vertical axis). *k*-mers are sorted according to their mapping location (in *Ace1* or elsewhere), and genomes are sorted according to their phenotype (resistant/susceptible). **D)** Pearson’s correlation coefficients (*r*) between *k-*mer frequency and number of *Ace1* copies in each genome (*n* = 439 significant *k-*mers). *k*-mers are coloured according to their mapping location (in *Ace1* or elsewhere) and sorted by the values of *r*.

We thus aimed to identify signals of selection in resistant *A*. *coluzzii* using the population branch statistic (*PBS*, Fig 5A), which identifies regions with an excess of genetic differentiation in a focal population (here, resistant Ivorian mosquitoes) relative to a basal level of differentiation between a closely related population (susceptible Ivorian mosquitoes) and a more distant control (a population of *A*. *coluzzii* from Angola; see Methods). *PBS* is a powerful test to detect recent selection acting on incomplete sweeps and standing variation [36,37], and is therefore well-suited to investigate a scenario—like ours—in which the population of interest has not yet diverged.

We estimated *PBS* along genomic windows to identify regions in which resistant Ivorian specimens had an excess of genetic differentiation (see Methods). In total, we identified 43 genes within six regions with excess differentiation in resistant *A*. *coluzzii* (*PBS* > 0, *FDR* < 0.001; see Methods; Fig 5A). This set of candidates contained eleven mostly uncharacterised genes located downstream of *Ace1* and within the duplicated region, two ionotropic receptors (*GLURIIb* and *GLURIIa*), and several proteases, among others (S7 Data). However, a detailed examination of haplotypes in these regions did not identify selection signatures specific to the resistant specimens, either as swept haplotypes or increased Garud *H*_*12*_ values (S16 Data). In fact, genetic differentiation between resistant and susceptible specimens in each of these genes was low (*F*_*ST*_ < 6% in all cases, including coding and non-coding variants; S7 Data). These results suggest that they are unlikely to be directly associated with the resistance phenotype. The *Ace1* gene itself was on the verge of the significance threshold (*FDR* = 3.2 × 10^−3^, *PBS* = 0.031) and exhibited low differentiation (*F*_*ST*_ = 1.2%). This apparent contradiction regarding the *Ace1* phenotypic association can be explained by the fact that pirimiphos-methyl resistance is caused by a combination of mutations—a SNP within a heterogeneous duplication—that is not captured by the diploid allelic frequencies used in *PBS* calculations [36].

To overcome this limitation, we performed a *k-*mer association study comparing the resistant and susceptible Ivorian *A*. *coluzzii*. *k*-mer association studies test for differential frequencies of tracts of nucleotides of length *k* in different groups of genomes, and are able to identify genetic variation patterns linked to both SNPs and structural variants, such as CNVs, without requiring a reference annotation [38]. We identified *ca*. 767 million different *k*-mers of length *k* = 31 bp across all 71 samples (see Methods). Among these, 9,603 *k*-mers were significantly enriched in resistant specimens (Spearman correlation test, *FDR* < 0.001). The 9,603 significant *k-*mers were assembled into 446 unique sequences composed of more than one *k*-mer (median length = 54 bp), which we then aligned to the *A*. *gambiae* reference genome. We retained sequences that could be mapped to chromosomes 2, 3 or X (409 out of 446) for further analysis (listed in S8 Data). Among these, the vast majority (*n* = 389, 95.1%) aligned to the *Ace1* duplicated region, while the remaining significant sequences aligned in scattered regions across the rest of the genome (*n* = 20, 4.9%; Fig 5B). Yet, 19 out of 20 *k-*mers that did not map to *Ace1* had a very similar frequency profile in resistant and susceptible samples than the 389 *Ace1*-linked *k*-mers, being absent and present in the same genomes (Fig 5C). In fact, the *k*-mer frequencies of these 19 sequences was strongly correlated with *Ace1* copy number (Pearson correlation *r* > 0 and *p* < 1 × 10^−4^; Fig 5D). This suggests that these 19 *k*-mers represent a non-independent signal that also reflects the association of *Ace1* mutations with resistance, and can be parsimoniously explained by non-specific sequence alignments (see Discussion). Only one remaining sequence did not correlate with *Ace1* copy number (Pearson correlation *r* < 0.0, maroon arrow in Fig 5C and 5D), indicating that it may represent another variant involved in resistance. The primary alignment of this sequence mapped to a non-coding region of chromosomal arm 2L (from 2L:8,662,023), with no proximity to any gene of known function, and did not exhibit signals of selection (S16 Data). Altogether, these results support the conclusion that *Ace1* mutations are the primary driver of pirimiphos-methyl resistance in this *A*. *coluzzii* population.

### Selection and introgression of *Ace1* duplications in *A*. *gambiae* and *A*. *coluzzii*

A high degree of geographical and phylogenetic overlap is evident between *Ace1* duplications and *280S* alleles across four countries (Côte d’Ivoire, Ghana, Burkina Faso and Guinea) and two different species (*A*. *coluzzii* and *A*. *gambiae*; Fig 2A). Previous studies provide two key insights to understand this pattern. First, *Ace1* duplications across West African populations have concordant breakpoints [30,34], which suggests they share a common evolutionary origin despite their multi-species distribution. Second, partial sequencing of *Ace1* has shown that *280S* alleles share highly similar haplotypic backgrounds in both West African *A*. *gambiae* and *A*. *coluzzii* [22,24,27], which is suggestive of inter-specific introgression and a selective sweep. The most parsimonious hypothesis linking our results and these observations would posit that (i) the high similarity of *280S*-carrying haplotypes across the *A*. *gambiae–A*. *coluzzii* species boundary is shared along the entire duplicated region (*ca*. 200 kbp); and (ii) this similarity is due to a selective sweep linked to *280S* and the duplication, which was followed by introgression between *A*. *gambiae* and *A*. *coluzzii*.

**Fig 6:**
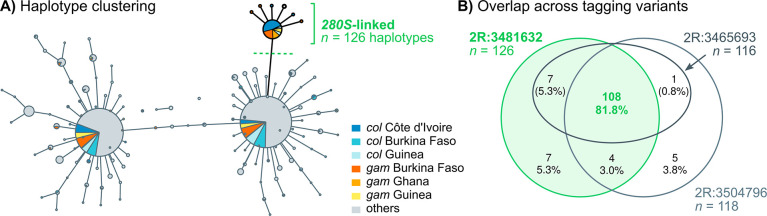
Haplotype clustering network around *Ace1*. **A)** Minimum spanning tree of haplotypes around the *280S-*linked variant 2R:3481632 (± 300 bp, *n* = 104 phased variants). Node size reflects number of haplotypes belonging to each cluster, which are color-coded according to species and geographical origin. Edges link haplotype clusters separated by one substitution. Singleton clusters have been removed from this view (see S10 Data). A cluster of identical haplotypes linked to the *280S-*tagging variant is highlighted in green. **B)** Venn diagram representing the overlap of samples belonging to the *280S-*linked haplotype clusters identified around each of the three tagging variants (S10 Data).

We tested this hypothesis by examining the profile of haplotype conservation and signatures of selection around the *Ace1* duplication. To this end, we first had to phase the *Ace1* duplication. However, accurate variant phasing within the duplication is confounded by the fact that this region is effectively polyploid in multiple samples, which results in uneven sequencing depth along this region (S17B Data) and a low density of phased variants (S9A and S9B Data). To circumvent these limitations, we scanned the duplication region to find tagging variants that (i) were in strong linkage disequilibrium with *G280S* and the duplication (Huff and Rogers’ *r* > 0.95; S9B Data, C); and (ii) belonged to diploid regions within the duplication (S9D–S9F Data). We found three tagging variants embedded in regions that fitted this definition, located within −26 kbp and +12 kbp of *G280S* (see Methods for details). We used these tagging variants to phase the duplication, building micro-haplotype networks (minimum spanning trees; Fig 6) and identifying sets of haplotypes in linkage disequilibrium with the *Ace1* mutations.

The minimum spanning tree built from haplotypes located around the first tagging variant (Fig 6A) identified a cluster of 126 identical haplotypes carrying the *280S*-linked allele (Fig 6A, labelled with green text) and two larger haplotype clusters linked to the *wt 280G* allele (Fig 6A). The *280S* cluster contains identical haplotypes from West African populations (Côte d’Ivoire, Burkina Faso, Ghana and Guinea) of both *A*. *coluzzii* and *A*. *gambiae*. On the other hand, *wt*-linked clusters have wider geographical distributions (including samples from West, Central and Eastern sub-Saharan Africa). The other three tagging variants show haplotype networks with a similar structure, with clusters of *ca*. 120 *280S*-linked identical haplotypes originating in the same *A*. *gambiae* and *A*. *coluzzii* specimens from West Africa (Fig 6B, S10 Data). The consistent clustering among the three tagging variants implies that these micro-haplotype networks reflect genetic signatures common across the duplicated region, and are therefore robust to the difficulties in phasing variants within a heterogeneous duplication (S9 Data). Thus, this result suggests that haplotype similarity between *A*. *gambiae* and *A*. *coluzzii* extends beyond the *Ace1* gene.

**Fig 7:**
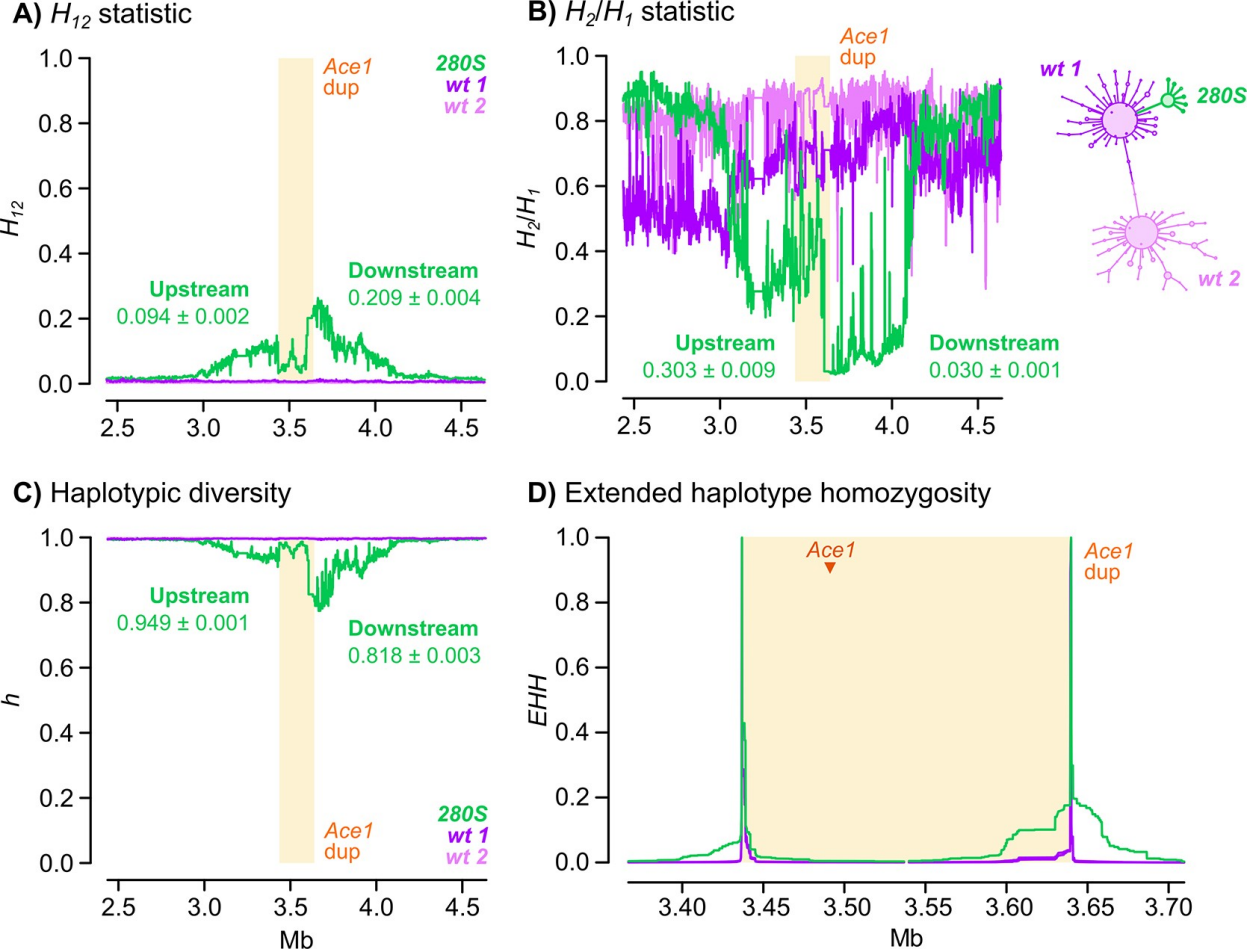
Positive selection around the *Ace1* duplication. **A-C)** Profile of Garud’s *H*_*12*_, *H*_*2*_*/H*_*1*_ and haplotypic diversity around the *Ace1* duplication, for haplotypes carrying the *280S*- or *wt*-tagging variants at 2R:3481632. Statistics are calculated in blocks of 500 variants with 20% block overlap. Includes averages of each statistic outside the duplication, upstream and downstream of the breakpoints (with standard errors from jackknife haplotype resampling). **D)** Extended haplotype homozygosity (*EHH*) at the duplication breakpoints for haplotypes carrying the *280S*- or *wt*-tagging variants at the 2R:3481632 locus. Additional plots for all tagging variants are available in S11 and S12 Data. Note that Garud’s *H* statistics and *EHH* assume a diploid genome, and estimates from within the duplication can thus be biased.

We also investigated possible signals of positive selection upstream and downstream of the *Ace1* duplication breakpoints using Garud’s *H* statistics, haplotypic diversity, and extended haplotype homozygosity (Fig 7, S11 and S12 Data). The profile of Garud’s *H*_*12*_ statistic in *280S-*linked haplotypes showed peaks both upstream and downstream of the *Ace1* duplication (Fig 7A), which coincided with a low *H*_*2*_*/H*_*1*_ ratio (Fig 7B) and low haplotypic diversity (Fig 7C), and is thus indicative of a hard selective sweep in this region [39,40]. Notably, the region of extended haplotype homozygosity associated with this sweep was still apparent upstream and downstream of the duplication breakpoints, i.e., far away from the focal tagging variant used to define *280S*-linked haplotypes (Fig 7D).

**Fig 8:**
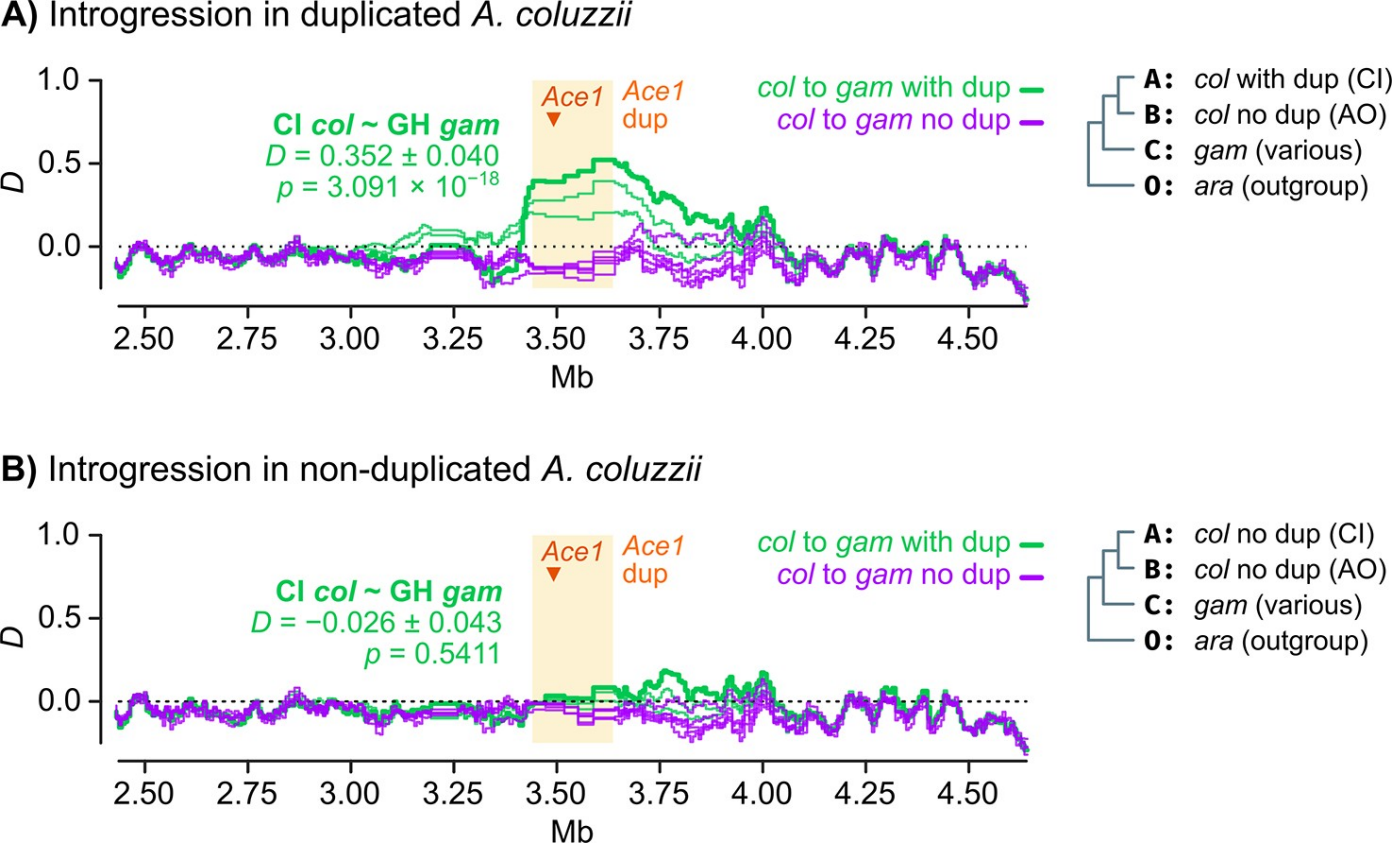
Introgression of the *Ace1* duplication. **A)** Profile of Patterson’s *D* statistic around the *Ace1* duplication, testing evidence of introgression between duplicated *A*. *coluzzii* from Côte d’Ivoire (population A) and various *A*. *gambiae* populations (population C) with or without duplications (green and purple lines, respectively). We used non-duplicated Angolan *A*. *coluzzii* as a contrast (population B) and *A*. *arabiensis* as outgroup (O). *D* is calculated in windows of 5,000 variants with 20% overlap, and the average value of D along the duplication region is shown for the Côte d’Ivoire *A*. *coluzzii* / Ghana *A*. *gambiae* comparison, with standard errors derived from block-jackknife. **B)** Id., but using non-duplicated *A*. *coluzzii* from Côte d’Ivoire as population A. Detailed lists of all statistical tests and replicate analyses with additional populations are available in S13 Data. Species abbreviations: *gam*, *A*. *gambiae*; *col*, *A*. *coluzzii*; *ara*, *A*. *arabiensis*. Country abbreviations: AO, Angola; CI, Côte d’Ivoire; GH, Ghana.

Next, we tested whether *280S* alleles and the duplication spread jointly between *A*. *coluzzii* and *A*. *gambiae* via introgression (Fig 8). We examined the chromosome-wide profile of introgression between *A*. *gambiae* and *A*. *coluzzii* populations using Patterson’s *D* statistic [41,42], which compares allele frequencies between three putatively admixing populations (A, B and C) and one outgroup (O); and can identify introgression between populations A and C (*D* > 0) or B and C (*D* < 0). Specifically, we tested whether duplicated *A*. *coluzzii* from West African populations (population A) introgressed with *A*. *gambiae* populations from West and Central Africa (C), using *wt* Angolan *A*. *coluzzii* as a control (B) and *A*. *arabiensis* as an outgroup (O).

We evaluated whether the *Ace1* duplication introgressed between *A*. *gambiae* and *A*. *coluzzii* in two scenarios: (i) in specimens with CNVs from populations with *Ace1* resistance mutations (Fig 8A), and (ii) in non-duplicated specimens from the same populations (Fig 8B). We only find evidence of introgression in the first case, i.e. between West African *A*. *gambiae* and *A*. *coluzzii* specimens carrying *Ace1* duplications (Fig 8A). For example, we found evidence of introgression between duplicated Ghanaian *A*. *gambiae* and duplicated Ivorian *A*. *coluzzii* (*D* = 0.352 +/- 0.040, *p* = 3.1 × 10^−18^ from a standard distribution of block-jackknifed estimates; green lines in Fig 8A); but not with non-duplicated Ivorian *A*. *coluzzii* (*D* = -0.026 +/- 0.043, *p* = 0.54; green lines in Fig 8B). In all comparisons where introgression was apparent, the genomic region of increased *D* values extended along the entire duplicated region. On the other hand, there was no evidence of introgression in any comparison involving non-duplicated *A*. *gambiae* (*D* ≈ 0; purple lines in Fig 8A and 8B).

**Fig 9:**
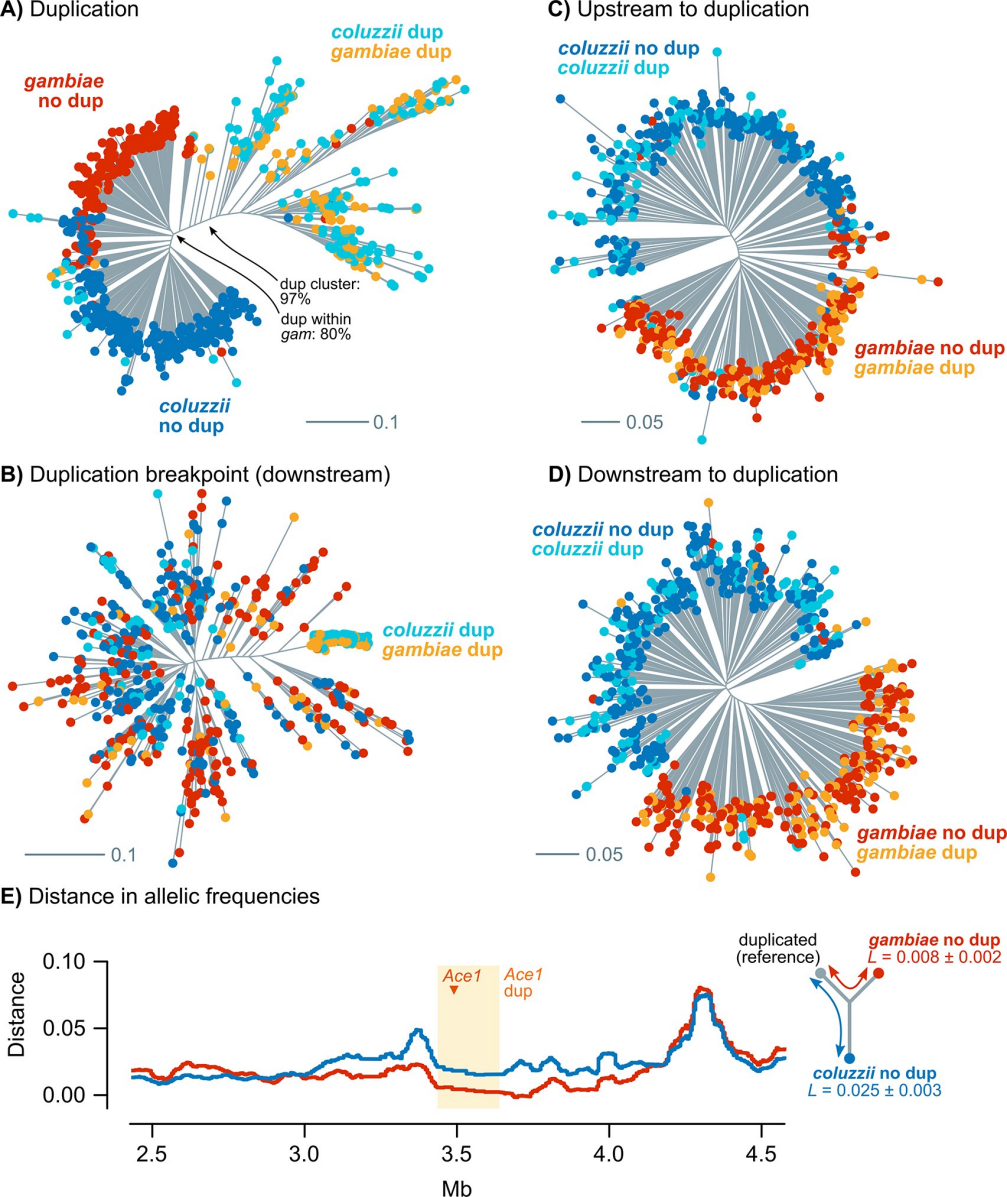
Phylogenetic analysis of introgression in *Ace1*. **A)** Maximum-Likelihood phylogenetic analysis of 690 Western African haplotypes from the *Ace1* duplicated region (2,787 phased variants), using a GTR model with ascertainment bias correction, empirical state frequencies and four Γ rate categories. **B-D)** Id., using variants beyond the downstream duplication breakpoint (512 variants) and 1Mb upstream and downstream of the duplication (3,935 and 3,302). Tips are color-coded according to species and duplication presence. Source alignments and complete phylogenies with supports in all nodes are available in S14 and S15 Data. **E)** Distance in allelic frequencies between groups of duplicated specimens and *wt A*. *coluzzii* and *A*. *gambiae*, calculated using the three-population branch statistic in windows of 5,000 variants along 2R (see Methods). Includes estimated branch lengths (*L*) from within the duplication region.

To establish the direction of introgression, we performed a phylogenomic analysis of haplotypes from the duplicated region (Fig 9). Firstly, haplotypes with *Ace1* duplications formed a single clade containing *A*. *gambiae* and *A*. *coluzzii* sequences to the exclusion of all non-duplicated sequences from both species. This clustering was also found in haplotypes located immediately downstream of the duplication breakpoint (Fig 9B, coinciding with the region of *D* > 0 in Fig 8A); but not in control phylogenies built from non-introgressed regions further upstream and downstream of the duplication (1 Mb away), which exhibited a topology with species-specific clades for both duplicated and non-duplicated specimens (Fig 9C and 9D). Secondly, we found that duplicated sequences were phylogenetically closer to the *wt A*. *gambiae* clade than to *wt A*. *coluzzii* (Fig 9A). Thirdly, an analysis of distance in allelic frequencies between *wt A*. *gambiae*, *wt A*. *coluzzii* and duplicated haplotypes also indicated that the duplicated haplotypes are more similar to *wt A*. *gambiae* than to *wt A*. *coluzzii*: the branch leading to *wt A*. *coluzzii* since the divergence from the duplicated specimens was longer (*L* = 0.026 ± 0.003 standard error) than the branch leading to *wt A*. *gambiae* (*L* = 0.008 ± 0.002, Fig 9E). This difference was also apparent when we analysed duplicated *A*. *coluzzii* and *A*. *gambiae* specimens separately (S18 Data), and extended immediately upstream and downstream to the duplication breakpoints (Fig 9E). Together, these results indicate that the duplicated haplotypes emerged from a *A*. *gambiae wt* background and later introgressed into *A*. *coluzzii*.

Altogether, these results show that the *280S* resistance alleles present across West Africa appear in similar genetic backgrounds irrespective of the sampled population and species, and that this similarity extends beyond the *Ace1* gene to encompass the entire duplicated region (Fig 6). The low haplotypic diversity is due to a hard selective sweep, detectable at the duplication breakpoints (Fig 7). This resistance haplotype, which spans *ca*. 200 kbp and includes *Ace1* and ten other genes (S3 Data) emerged in *A*. *gambiae* and later spread to *A*. *coluzzii* (Figs 8 and 9).

## Discussion

### Evolutionary history of *G280S* and *Ace1* duplications

Our results show that pirimiphos-methyl resistance is associated with a combination of two mutations in *Ace1*: the *G280S* SNP and CNVs. In West Africa, virtually all CNVs are found as duplications of a wide region (*ca*. 200 kbp) that includes *Ace1* and 10 other genes [30,34]. This duplication has a unique evolutionary origin in the *A*. *gambiae*/*A*. *coluzzii* species pair, as its breakpoints are consistent in all populations from both species studied so far [30,34]. The distribution of duplication-*280S* resistance haplotypes observed in the 1000 Genomes cohort can be explained by three evolutionary events: the *G280S* mutation, an internally heterogeneous duplication, and inter-specific introgression. Furthermore, recent sampling efforts have identified internal deletions within the duplication in both *A*. *gambiae* and *A*. *coluzzii*, which appear to be increasing in frequency [31]. Homogeneous duplications carrying only *280S* alleles are rare in the 1000 Genomes dataset but appear to be quite common in some *A*. *gambiae* populations, though less so in *A*. *coluzzii* (Table 2; [24,31]).

At present, we cannot establish the relative order of occurrence of the SNP and CNV in *Ace1* because these mutations are tightly linked in the 1000 Genomes dataset (Fig 2A). Nevertheless, the detection of *wt* homogeneous duplications in samples collected in 2002 in southern Ghana [24]—a country where *280S* alleles have more recently been found at high frequencies (Fig 1)—raises the possibility that the SNP might have occurred on an already duplicated background. This order of events would have initially resulted in heterogeneous duplications, providing resistance and reducing the fitness costs associated with impaired acetylcholinesterase activity in the absence of insecticide [19,29]. If that were the case, the emergence of *280S-*homogeneous duplications could have occurred via any of the following mechanisms: (i) a secondary loss of a *wt* copy; (ii) a parallel *G280S* mutation replacing the *wt* nucleotide in the initially heterogeneous duplication; (iii) or double recombination or gene conversion events involving the *280S-*carrying copy.

After these sequential mutations, the joint duplication-*280S* resistance haplotype introgressed from *A*. *gambiae* into *A*. *coluzzii* (Figs 8 and 9). This *A*. *gambiae* origin is consistent with earlier studies of *Ace1* variation in West African locations, which generally reported higher frequencies of *280S* [20,22,27] and CNVs [31] in *A*. *gambiae* than in *A*. *coluzzii*, as one would expect if they had a longer evolutionary history in the former. Whilst previous studies had suggested that the similarity of *280S* haplotypes in *A*. *coluzzii* and *A*. *gambiae* was due to inter-specific introgression [22,24,27], they were focused on sequencing of the *Ace1* gene and could not assess the relationship between introgression and the wider duplicated region (*ca*. 30 times longer than the *Ace1* coding region). Furthermore, analyses focused on the *Ace1* gene body are confounded by the lack of sequence variation in this region (S9 Data). By leveraging variation in linkage disequilibrium beyond the gene, we are able identify clear signatures of introgression along the entire *Ace1* duplication region (Fig 8).

### Genetic basis of pirimiphos-methyl resistance in *A*. *coluzzii* and *A*. *gambiae*

We have uncovered a strong association between resistance to pirimiphos-methyl and the possession of two or more copies of the *Ace1 280S* allele in *A*. *coluzzii* from Côte d’Ivoire (Fig 3). While the *G280S* mutation alone has been previously linked to resistance to carbamates and organophosphates in multiple insects [13–15,19,27,43–45], our results indicate that resistance to pirimiphos-methyl specifically relies on the presence of multiple *280S* alleles.

Across the 1000 Genomes cohort, specimens with *280S* alleles can be grouped into two categories according to the number of *Ace1* copies (Fig 2C): (i) heterogeneous duplications with multiple *280S* alleles and/or multiple *wt*, and (ii) high-copy, *280S*-homogeneous duplications. Heterogeneous duplications are the most frequent combination of *Ace1* mutations (113 out of 119 samples, Fig 2B) and the only one identified in the *A*. *coluzzii* population from Côte d’Ivoire (Tiassalé). We next examined the contribution of *280S* and duplications to pirimiphos-methyl resistance in a wider array of *A*. *gambiae* and *A*. *coluzzii* populations from Ghana, Côte d’Ivoire, and Togo (Tables 1 and 2). We identified a common theme in most populations surveyed here: (i) a combination of *wt* and *280S* alleles was the most common resistant genotype in seven out of nine populations analysed, including all of the *A*. *coluzzii* sampling locations and three out of five in *A*. *gambiae* (Table 1); and (ii) the ratio of *280S* alleles and *Ace1* copy number were significantly associated with resistance in six out of eight tested populations (Table 2; note that low sample size precluded this analysis in the ninth population). This provides a wider demonstration that the combined effect of *G280S* and *Ace1* duplications is significantly associated with pirimiphos-methyl resistance across multiple West African populations of both species; consistent with the requirement for multiple copies of *280S*—shown in the our Tiassalé population analysis (Fig 3B)—to produce a resistant phenotype.

We also identified six genomes with high-copy, *280S*-homogeneous loci sampled from the Ghanaian *A*. *gambiae* population in the 1000 Genomes dataset (Figs 1 and 2). These samples had not been assayed for pirimiphos-methyl resistance prior to sequencing. However, our extended genotype-phenotype analysis in nine West African populations (Table 1) showed that (i) *280S* homozygotes exhibited increased resistance to pirimiphos-methyl, and that, in fact, they were the most abundant resistance allele in two *A*. *gambiae* populations (Baguida and Obuasi). It is worth mentioning that survival rates among *280S* homozygotes were noticeably lower these two locations (56.8% - 58.9%) than in populations where specimens with both *wt* and *280S* alleles were more common (> 95%; Table 1). Our analyses cannot fully capture the resistance landscape in these populations, because we have focused on the role of *280S*-heterogeneous duplications (as they were the most abundant resistant type in the 1000 Genomes cohort; Fig 1). Thus, we cannot presently explain these differences, which may reflect an effect of different phenotyping strategies employed in each population (see Methods), or additional resistance mechanisms. A detailed examination of *280S*-homogeneous duplications—e.g. effects on gene dosage or fitness, as performed on heterogeneous duplications in *Culex pipiens* [46]—could shed light onto the phenotypic variability observed in populations with high penetrance of *Ace1* mutations.

Finally, we have performed a genome-wide scan in a population of *A*. *coluzzii* from Côte d’Ivoire (Tiassalé) to identify loci associated with pirimiphos-methyl resistance (Fig 5). Our investigation of the association between *k*-mer frequencies and resistance confirmed the overwhelming concentration of phenotypic association in the *Ace1* duplication (Fig 5B), supporting the conclusion that it is the primary driver of resistance in this population. The correlations between *Ace1* duplication and all but one of the *k*-mers significantly associated with resistance, but mapping elsewhere in the genome strongly suggests non-independence of the signals, but the exact cause of this is unclear. Whilst we cannot entirely discount linkage disequilibrium arising from epistasis, a simpler and parsimonious explanation would be that this result is caused by non-specific or incorrect sequence alignments, possibly associated with currently unrecognised variation in the *Ace1* duplication.

The lack of clear signals of pirimiphos-methyl adaptation other than *Ace1* in this population may reflect its collection at an early stage of the development of pirimiphos-methyl resistance in West Africa [47]. Therefore, our genome-wide analyses would likely only capture pre-existing variants that were enriched in samples resistant to other insecticides. *Ace1* mutations and duplications, which cause cross-resistance with previously employed pesticides [22,24,27–31,48], fit this definition and are sufficient to produce significant resistance. However, future analyses on recent collections from populations subjected to regular pirimiphos-methyl-based IRS may reveal additional mechanisms more specifically selected by this insecticide.

### Implications for insecticide intervention programmes

Our results demonstrate that the duplication-*280S* haplotype represents a powerful marker to diagnose resistance to pirimiphos-methyl in *A*. *coluzzii* and *A*. *gambiae*, permitting the spread of resistance to be tracked from any preserved sample from which DNA can be extracted. Our initial survey of an *A*. *coluzzii* population from Tiassalé (Côte d’Ivoire) shows that discrimination between susceptible and resistant specimens can be attained with high accuracy in this population according to the number of *280S* alleles. Specifically, following the resistance thresholds apparent in Fig 3B, we can (i) classify *wt* homozygous samples as susceptible (100% predictive value) and (ii) separate individuals with alleles between those with more *wt* than *280S* alleles (susceptible) and those with equal or more *280S* than *wt* alleles (resistant). This would yield a positive predictive value of 77% for resistance, and a negative predictive value of 85% for susceptibility.

In practice, precise quantification of the number of resistant *280S* alleles will often be difficult. Nevertheless, we show that key variation in *Ace1* can be captured using either of two qPCR genotyping assays that predict pirimiphos-methyl resistance in populations of *A*. *gambiae* and *A*. *coluzzii* (Table 2): (i) a measurement of the ratio of *280S* to *280G* alleles [35], and (ii) the number of *Ace1* copies [28]. Crucially, measurements of the *280S* allelic ratio can be obtained from the first assay, which is currently widely used for genotyping of the *G280S* mutation [35]. This provides good diagnostic capacity not only in specimens with *280S*-homogeneous duplications, but also in those with heterogeneous duplications, by scoring of the *280G/S* allele balance. The phenotypic assays we employed were intended to provide more accurate separation than standard dose approaches [49], but the strong effect of *Ace1* CNVs and *G280S* mutations on pirimiphos-methyl resistance is sufficiently robust for resistance diagnosis in standard discriminating dose assays as well–as used in the Tiassalé *A*. *coluzzii* specimens from the 1000 Genomes cohort.

Furthermore, monitoring studies must note that this resistance haplotype is still evolving, especially in the light of the fitness trade-offs associated with *Ace1* resistance alleles–namely, that heterogeneous duplications offset the fitness cost of *280S* alleles in the absence of insecticide exposure [19,29], and that variable copy number can result in gene dosage disturbances [30]. A recent survey has shown that internal deletions within the duplicated region, downstream to *Ace1*, are spreading in both *A*. *gambiae* and *A*. *coluzzii* [31]. The authors have proposed that these deletions reduce the fitness costs of duplications by ameliorating deleterious changes in gene expression [31]. These small deletions are absent from the Ivorian *A*. *coluzzii* genomes analysed here [34], but future genome monitoring studies could investigate their spread and identify signals of selection associated with their proposed selective advantage. Likewise, the independently evolved *Ace1* CNV that we have identified in one *A*. *gambiae* specimen from Guinea (Fig 2B, S4 Data) might merit further attention in the future. Finally, as noted above, we cannot rule out the possibility that, following several years of widespread use of pirimiphos-methyl for IRS, additional non-target site-based resistance mechanisms might have emerged.

The duplication-*280S* haplotype pre-dates the widespread adoption of pirimiphos-methyl in 2013 [1], which indicates that it likely spread due to its adaptive value with respect to previously employed insecticides used in disease control or agriculture, such as carbamates (e.g. bendiocarb [28,30]) or other organophosphates (e.g. fenitrothion, chlorpyrifos-methyl [22,30]). In that regard, we show that the evolution of the pirimiphos-methyl resistance haplotype is deeply intertwined with that of previous insecticide adaptations, and reveal how continued genomic monitoring studies can help us understand the influence of previous interventions on future population control efforts. An unsuccessful small-scale trial of prototype pirimiphos-methyl wall hangings—interestingly also conducted in Tiassalé at a similar time to collection of our samples—documented significantly greater survival of Ace1 *280S* carriers (primarily heterozygotes) than *wt* [23]. However, operational impacts of the *Ace1* mutations have yet to be demonstrated and investigation represents a high priority.

Whilst efficacy of pirimiphos-methyl in Actellic IRS formulations appears to have been retained, at least until 2017 [5], its sustainability could be limited in West African *Anopheles* populations where resistance is already common (Figs 1 and 2). This emphasises the crucial importance of currently-commencing resistance management strategies rotating pirimiphos-methyl spraying with additional IRS insecticides with alternative modes of action [50]. Our demonstration of the utility of *Ace1*-based diagnostics should aid in molecular surveillance to support these insecticide resistance management programmes.

## Methods

### Data collection

We used genomic variation data from individual mosquitoes, obtained from the Phase 2 (AR1) of the *Anopheles gambiae* 1000 Genomes project, as described in [32,33]. This dataset consists of 1,142 wild-caught mosquitoes (1,058 females and 84 males) from 33 sampling sites located in 13 sub-Saharan African countries (listed in S4 Data), and collected at different times between 2009 and 2012 (with the exception of samples from Gabon and Equatorial Guinea, collected in 2000 and 2002 respectively).

A detailed explanation of the methods used in whole-genome sequencing and variant calling can be found in the original publications [32,33]. Briefly, DNA from each specimen was extracted with a Qiagen DNeasy Blood and Tissue Kit (Qiagen Science, US) and sequenced with the Illumina HiSeq 2000 platform (Wellcome Sanger Institute, UK) using paired-end libraries (100 bp reads, 100–200 bp insert sizes), aiming at a 30× coverage per specimen. Variant calling was performed using *bwa* [51] 0.6.2 and the *GATK* 2.7.4 *UnifiedGenotyper* [52]. Haplotype phasing was estimated with *SHAPEIT2* [53], and variant effects were predicted using *SnpEff* 4.1b [54]. We downloaded the genotype calls, SNP effect predictions, and haplotype phasing as available in *Anopheles gambiae* 1000 Genomes Phase 2 online archive [55].

The catalog of *Ace1* CNVs in the 1000 Genomes dataset (Phase 2) was obtained from [34]. There, the authors calculated the coverage (sequencing depth) of each whole-genome sequenced sample in non-overlapping windows along the genome (300 bp) and normalised this value so as to obtain an expected average value of coverage = 2 in non-duplicated, diploid regions. A Gaussian HMM procedure was then applied to the normalised windowed coverage data so as to call windows with heightened normalised coverage (>2). A detailed account of these methods can be found in the original publication [34]. The duplication calls, breakpoint sequences and per-window coverage calculations at the *Ace1* region are available in S4 Data and S17 Data.

We retrieved the reference genome information for *A*. *gambiae* from VectorBase [56], including the genome assembly (PEST, AgamP4 version), gene annotation coordinates in GFF format (AgamP4.9) and gene names and descriptions.

### Genotype-phenotype association in Côte d’Ivoire *A*. *coluzzii* genomes

As part of *Anopheles gambiae* 1000 Genomes Phase 2, 71 *A*. *coluzzii* specimens from Côte d’Ivoire were collected and phenotyped for pirimiphos-methyl resistance before whole-genome sequencing [32]. These samples were collected as larvae in rice fields near Tiassalé (coordinates: -4.823, 5.898) between May and September 2012, and were identified as *A*. *coluzzii* using a PCR assay [57]. The 71 larvae were tested for resistance to pirimiphos-methyl resistance using a WHO tube assay with 0.25% impregnated papers [58], which led to the identification of 31 resistant (live) and 40 susceptible (dead) specimens.

We tested the association of these resistance phenotypes with various genetic variants present in *Ace1* in this population. This includes the non-synonymous mutations *G280S* and *S65A*, the number of *Ace1* copies, and the number of *280S* alleles in each individual sample (listed in S6A Data).

For the non-synonymous mutations, we assessed genotype-phenotype associations for each variant independently using Fisher’s exact test (*gmodels R* library [59]) and estimated odds ratio and 95% confidence intervals using the Woolf method with Haldane-Anscombe correction (*Prop*.*or* function in the *pairwiseCI R* library [60]). For the *Ace1* and *280S* copy numbers, we used used binomial generalised linear models (*glm* function in *R stats* library, family *binomial*) to obtain odds ratio estimates for each of the four variables.

We also built a binomial generalised linear models with all four variables (*G280S* and *A65S* genotypes; *Ace1* and *280S* copy number) and a step-wise procedure to remove non-informative variants from the model (*step* function in *R stats* using *k = log(n)* as the degree of freedom, as required by the Bayesian Information Criterion; *n* represents the number of samples in our dataset). The number of *Ace1* copies and the number of mutated alleles were encoded as continuous variables, and the genotypes of *G280S* and *S65A* were encoded as categorical variables. The performance of all generalised linear models was assessed relative to a null model (intercept as the only variable) using a *χ*^*2*^ test in an analysis of variance (*anova* function in the *R stats* library). A detailed statistical analysis of all comparisons mentioned above can be found in S6A and S6B Data.

The number of *Ace1* copies in each genome of *Anopheles gambiae* 1000 Genomes Phase 2 was retrieved from [34]. In that study, copy number states of multiple CNVs were inferred using a HMM-based predictive model that took as input the normalised sequencing depth calculated along non-overlapping 300 bp genomic windows.

The estimated number of *280S* alleles in each genome was estimated in the following manner: (i) we retrieved the copy numbers of *Ace1* in each genome (*C*; see above); (ii) we calculated the fraction of reads supporting the reference and alternate alleles; and (iii) assigned the number of *280S* alleles (*S*) as the value that minimised the difference between the fraction of alternate alleles and *S/C*, for all discrete *S* values between 0 and *C*. For example, a genome with three *Ace1* copies (*C* = 3) and 30% of reads supporting *280S* would carry one *280S* allele (*S* = 1), given that *S/C* = 1/3 ≈ 30%.

### Genotype-phenotype association in additional West African populations

We collected 1080 female specimens of two species (*A*. *coluzzii* and *A*. *gambiae*) from six different locations across West Africa (Côte d’Ivoire: Aboisso; Ghana: Madina, Korle-Bu, Weija and Obuasi; Togo: Baguida). Species identity was determined using two methods designed to discriminate between *A*. *gambiae*, *A*. *coluzzii* and *A*. *arabiensis*: a PCR of species-specific SINE insertion polymorphisms as described in [61], and a high-resolution melt curve analysis [62]. Details for each of these methods, including primer sequences, are available in S6G Data. The complete list of specimens, sampling times and locations, and species identification are available in S6C Data and S6E Data.

All 1080 specimens were phenotyped for pirimiphos-methyl resistance using a custom dose-response assay with WHO standard tubes [58]. For the Aboisso (Côte d’Ivoire), Korle-Bu, Weija and Madina samples, resistant specimens were determined as surviving a threshold concentration of pirimiphos-methyl after exposure during the larval stage, and susceptible specimens were identified as dead after a lower threshold dose (constant exposure time of 60 minutes). The exact concentration thresholds for the dose-response assay were determined on a per-site basis, tailored to the observed levels of resistance in each population and collection logistics, and are listed in S6C Data. Out of 1080 specimens, 951 could be assigned to one of the following populations, defined as combinations of species and collection locations with determined resistance phenotypes: *A*. *coluzzii* from Aboisso, Côte d’Ivoire (*n =* 55); *A*. *coluzzii* from Korle-Bu (*n* = 214), Madina (*n* = 42), and Weija (*n* = 131), Ghana; *A*. *gambiae* from Aboisso, Côte d’Ivoire (*n =* 82); *A*. *gambiae* from Baguida, Togo (*n* = 102); *A*. *gambiae* from Madina (*n* = 172), Obuasi (*n* = 140) and Weija (*n* = 13), Ghana.

For each sample, we determined the *G280S* genotype using a qPCR TaqMan assay as described by Bass *et al*. [35] (list of samples in S6C Data), in which *280S*- and *280G*-specific primers where labeled with different fluorescent dyes (FAM and HEX, respectively). We also calculated the ratio of FAM-to-HEX fluorescent dye signal in hetrerozygotes, which label *280S* and *280G* alleles respectively, as an index of the fraction of *280S* allele copies [28,48]. Detailed methods for these two genotyping assays are available in S6G Data.

We assessed the association of *G280S* mutations with resistance for each of the populations listed above using generalised linear models (*glm* function in *R stats* library, *binomial* family). The results from these tests are available in S6D Data.

A sub-set of these specimens (*n* = 201; listed in S6E Data) were also genotyped for CNV polymorphisms in the *Ace1* locus using a qPCR assay and a combination of primers for *Ace1* and two non-duplicated control genes (the *CYP4G16* gene AGAP001076 and the elongation factor AGAP005128), and were measured relative to values in *A*. *gambiae* specimens from the *wt* Kisumu colony (non-duplicated). Detailed methods for CNV genotyping assay are available in S6G Data and [28]. These samples were selected from the subset of specimens with both *280S* a*nd wt* alleles, in order to investigate the effect of both CNVs and fraction of 280S al*lele*s on resistance.

We used generalised linear models (*glm* function in *R stats* library, *binomial* family) to assess the effect of these two variables within each of the eight populations with available CNV data (four from *A*. *coluzzii*, four from *A*. *gambiae*; listed in Table 2 and S6F Data). Then, we obtained the minimal significant model for each population using a stepwise reduction procedure and the BIC criterion (*step* function in the *R stats* library), and assessed their fit against a null model (intercept as the only variable) with an analysis of variance and a *χ*^*2*^ test (*anova* function in *R stats*). The results from these tests are available in S6F Data.

### Haplotype clustering and analysis of selection signals in *Ace1*

Haplotypes along chromosome arm 2R were reconstructed using the dataset of phased variants from the *Anopheles gambiae* 1000 Genomes Phase 2 data. Specifically, we retained phased variants that were biallelic, non-singletons and segregating in at least one population. It must be noted that phased variants are scarce in the *Ace1* region (S9A and S9B Data) because the phasing procedure used in the original dataset can only be applied to diploid genomic regions [32,33], which is generally not the case along the *Ace1* duplication. In particular, phased variants had to be (i) biallelic and (ii) belong to an accessible genomic region, defined as having even sequencing coverage across samples and not having ambiguous alignments in more than one specimen. Yet, a duplicated region can still encompass short segments of diploid sequences, either due to internal deletions, as found in *Ace1* [31]); or to high divergence in one of the paralogous sequences, which results in only reads from the non-diverged (*wt*) paralog mapping to the reference genome and being phased as diploid.

We identified chromosomes carrying *Ace1* duplications that could be used for further analyses of selection signatures. To do so, we calculated the linkage disequilibrium between position *G280S* (itself tightly linked to the duplication) and all variants located between positions 3416800 and 3659600 along 2R (i.e., 20kbp upstream and downstream of the duplication breakpoints [3436800–3639600]). Linkage disequilibrium was estimated from genotype counts in each sample using Rogers’ and Huff *r* correlation statistic [63] as implemented in *scikit-allel* v1.1.10 library (*rogers_huff_r*) [64]. Then, we retrieved all variants with *r* > 0.95 that were (i) present in the subset of phased variants and (ii) were embedded within genomic regions that were, on average, represented by two haplotypes across samples (i.e. diploid). Three tagging variants fitted this definition (S9 Data): they were located in intergenic regions −26 kbp and +12 kbp of *G280S*, had minor allele frequencies similar to that of *280S* in Côte d’Ivoire (43%), were embedded in genomic windows that were effectively diploid (haplotype score *≈* 2; S9D–S9E Data, calculated with *GATK Unified Genotyper* in [32]), and had lower normalised sequencing depths across samples than the *Ace1* gene locus (S9F Data and S4B Data; coverage *≈* 2; data from [34]). These properties imply that it is possible to recover diploid haplotypes around the tagging variants.

We retrieved haplotypes surrounding each of the three tagging variants and clustered them by similarity using minimum spanning trees. Specifically, we retrieved phased variants ± 300 bp of each tagging variant (retaining variants that were biallelic, non-singletons and segregating in at least one population; these regions contained between 67 and 164 phased variants depending on the analysis; S10 Data). We used allele presence/absence data from each haplotype to build minimum spanning trees of haplotypes based on Hamming distances (breaking edges for distances >1). This distance matrix was then used to build medium spanning trees (*minimum_spanning_tree* function in *SciPy* 1.1.0 Python library [65], from the *sparse*.*csgraph* submodule). Tree visualizations were produced using the *graphviz* 2.38.0 Python library [66] and the *hapclust* library [67,68], and clusters of haplotypes were color-coded according to the population of origin or the presence/absence of the alternate allele (S10 Data).

We used these trees to identify groups of highly similar haplotypes linked to the *280S* or *wt* alleles in *Ace1* (S11 Data). We calculated the profile of Garud’s *H* statistics (*moving_garud_h* function, *scikit-allel*) [39,40] and haplotype diversity (*moving_haplotype_diversity* in *scikit-allel*) around the *Ace1* duplication region in windows of consecutive variants (500 variants with 20% overlap). These calculations were performed separately for the main groups of identical haplotypes (*280S-*linked or *wt-*linked) as identified around each of the tagging variants (minimum size = 100 haplotypes). In addition, we calculated the averages of these same statistics in the regions immediately upstream and downstream of the duplication breakpoints (2R:3436800–5000 bp and 2R:3639600 + 5000 bp, respectively), and we calculated standard errors of these estimates using sample jack-knifing.

Finally, we calculated the rates of extended haplotype homozygosity decay for each cluster (S12 Data), using 10,000 phased variants upstream and downstream of the duplication breakpoints (*ehh_decay* function in *scikit-allel*).

### Introgression analysis

We used Patterson’s *D* statistic [41,42] to detect introgression between *A*. *coluzzii* and *A*. *gambiae*. We retrieved the allele frequencies of all biallelic, non-singleton variants from chromosome arm 2R that were segregating in West African populations where we had identified *Ace1* duplications (namely: 75 *A*. *coluzzii* from Burkina Faso, 55 from Ghana, and 71 from Côte d’Ivoire; and 92 *A*. *gambiae* from Burkina Faso, 12 from Ghana and 40 from Guinea; total *n* = 345 genomes) [34], as well as Central African populations that we used as non-admixed controls (78 *A*. *coluzzii* from Angola; 69 *A*. *gambiae* from Gabon and 297 from Cameroon). In addition, we retrieved the genotypes at the same variant positions for the populations of *A*. *arabiensis* (12 genomes), *A*. *quadriannulatus* (10), *A*. *merus* (10) and *A*. *melas* (4) populations analysed in [69], which we used as outgroups in the calculation of Patterson’s *D*. In total, we retained *ca*. 12% of the 47,817,813 variants in 2R in each analysis.

To determine whether duplicated and non-duplicated populations had different introgression patterns in *Ace1*, we calculated windowed means of Patterson’s *D* (length 5,000 variants, 20% step; *moving_patterson_d* in *scikit-allel*) using various combinations of populations with and without the *Ace1* duplication (Fig 5A). This test requires genotype frequencies in four populations (two ingroups A and B; one candidate donor/receptor C with which A or B might have introgressed; and one unadmixed outgroup O) branching in a (((A,B),C),O) topology (Fig 5A). We observed the following convention: (i) we used West African *A*. *coluzzii* populations as A, discriminating between duplicated and non-duplicated subpopulations; (ii) we used non-duplicated *A*. *coluzzii* from Angola as control B; (iii) we used various *A*. *gambiae* populations as C, again analysing duplicated and non-duplicated specimens within each population separately; and (iv) *A*. *arabiensis*, *A*. *quadriannulatus*, *A*. *merus* and *A*. *melas* as outgroup O.

For each combination of (((A,B),C),O) populations, we calculated the average *D* statistic within the duplicated (position 3,436,800 to 3,639,600, spanning 202.8 kbp around *Ace1*), and estimated its deviation from the null expectation (no introgression: *D* = 0) with a block-jackknife procedure (block length = 100 variants; *average_patterson_d* function in *scikit*-*allel*), which we used to estimate the standard error, *Z-*score and the corresponding *p* value from the two-sided normal distribution (S13 Data).

In parallel, we performed a phylogenomic analysis of the reconstructed haplotypes of the *Ace1* CNV region (position 3436800 to 3639600) in the West African populations where duplications had been identified (75 *A*. *coluzzii* from Burkina Faso, 55 from Ghana, and 71 from Côte d’Ivoire; and 92 *A*. *gambiae* from Burkina Faso, 12 from Ghana and 40 from Guinea; total *n* = 345 genomes, 690 haplotypes). Specifically, we built an alignment of phased variants along the duplication (2,787 positions) and computed a Maximum-Likelihood phylogenetic tree using *IQ-TREE* 1.6.10 [70]. We used the GTR nucleotide substitution model with ascertainment bias correction, empirical state frequencies observed from each alignment, and four gamma distribution (*Γ*) categories (*GTR+F+ASC+G4* model in *IQ-TREE*). This model was selected by the *IQ-TREE* implementation of *ModelFinder* [71] (*TEST* mode) as the best-fitting model according to the BIC criterion. The best-fitting tree was found after 22,000 iterations (correlation coefficient threshold = 0.99). We estimated node statistical supports from 1,000 UF bootstraps [72,73]. Using the same approach, we built three additional phylogenies from genomic regions located immediately downstream of the duplication breakpoint (5 kbp, 512 variants), as well as upstream and downstream of the duplication breakpoints (3,935 and 3,302 variants extracted from 50 kbp segments located −1Mb and +1Mb), finding the best-fitting trees after 5,500, 7,659 and 27,400 iterations, respectively (correlation coefficient threshold = 0.99). The resulting phylogenetic trees were plotted, unrooted, using the *phytools* 0.6–60 [74] and ape 5.3 libraries (*plot*.*phylo*) [75]. Phylogenies and source alignments are available as S14 Data and S15 Data.

We also used allelic frequencies within the inversion to estimate the divergence times between specimens carrying the duplication (both *A*. *gambiae* and *A*. *coluzzii*), *wt A*. *coluzzii* and *wt A*. *gambiae*. This three-way calculation of divergence times reflects the amount of allele frequency change between one of the three groups relative to the other two, and is therefore analogous to the population branch statistic [36,76]. Following this logic, we estimated separately (i) the branch length of *wt A*. *coluzzii* relative to the separation of *wt A*. *gambiae* and the duplicated sequences, and (ii) the branch length of *wt A*. *gambiae* relative to the separation of *wt A*. *coluzzii* and the duplicated sequences. We repeated these analyses using only duplicated specimens from either species. We performed these calculations in non-overlapping windows of 100 variants with the *pbs* function in *scikit-allel*, and estimated standard errors using a block-jackknifing procedure. We used windows of 5,000 variants at 10% steps for visualisation (Fig 9E and S18 Data).

### Genetic differentiation in *A*. *coluzzii* from Côte d’Ivoire

We assessed the degree of genetic differentiation between resistant and susceptible *A*. *coluzzii* from Côte d’Ivoire. We focused on non-singleton variants that were segregating in this population. In total, 8,431,869 out of 57,837,885 (14.57%) variants from chromosomes 2, 3 and X were retained for further analysis.

For each chromosome arm (2R, 2L, 3R and X), we calculated genotype counts in each subpopulation (resistant and susceptible), and calculated their genetic differentiation using the Hudson’s *F*_*ST*_ statistic [77,78] along non-overlapping windows of 1,000 variants (*moving_hudson_fst* function in the *scikit-allel* v1.1.10 library [64] from Python 3.4). We also calculated the average *F*_*ST*_ in each chromosomal arm, with standard errors obtained from a block-jackknife procedure (using non-overlapping windows of 1,000 variants; *average_hudson_fst* function).

We calculated the normalised population branching statistic (*PBS*) [36,37] in non-overlapping windows of 1,000 variants along each chromosomal arm (*pbs* function in *scikit-allel*). using resistant and susceptible Ivorian *A*. *coluzzii* as test populations, and *A*. *coluzzii* from Angola as an outgroup. Angolan *A*. *coluzzii* (*n* = 78) where selected as outgroup due to their relative isolation relative to West African *A*. *coluzzii* populations [32,33] and their putatively naive profile of organophosphate resistance [1,4]. The distribution of *PBS* estimates along each chromosomal arm was standardised to unit variance (*standardize* function in *scikit-allel*), and the resulting distribution of *Z-*scores was used to derive a two-sided *p*-value that reflected extreme values of *PBS* (highly positive or negative). We corrected for multiple testing using local estimation of false discovery rates (*fdrtool* function in the *fdrtool* 1.2.15 *R* library [79]). Finally, we selected genomic windows with high differentiation in resistant specimens (standardised *PBS* > 0, significance threshold *FDR* < 0.001). To further characterise these *PBS* peaks, we (i) calculated Hudson’s *F*_*ST*_ in each genomic window (S7 Data); (ii) built neighbour joining phylogenies from the phased variants within each peak, to identify swept haplotypes (S16A Data; *nj* tool in the *ape R* package [75]); and (iii) calculated the profile of Garud’s *H*_*12*_ in the neighbouring regions, for resistant and susceptible specimens separately (S16B Data).

We performed a principal component analysis of resistant and susceptible Ivorian *A*. *coluzzii*. We first obtained a set of genetically unlinked variants from chromosomal arms 3R and 3L (so as to avoid the confounding effects of chromosomal inversions in arms 2R, 2L and X). We discarded linked variants within 500 bp consecutive windows (using a 200 bp step), using a Rogers and Huff’s *r* threshold value of 0.1 (*locate_unlinked* function in *scikit-allel*), and repeated this process for ten iterations for each chromosome arm. This filtering procedure resulted in 791 genetically unlinked variants from both chromosomal arms. We used the alternate allele count in each variant to construct a PCA using singular value decomposition (*pca* function from *scikit-allel*), and scaling the resulting coordinates using Patterson’s procedure [80].

### *k*-mer enrichment analysis in *A*. *coluzzii* from Côte d’Ivoire

We obtained *k*-mer counts for each of the 71 *A*. *coluzzii* samples using the *count* function in *jellyfish* v. 2.2.10 [81], using a *k* = 31 bp (parameters: *-C -m 31—out-counter-len 2 -s 300M —bf-size 10G*). To reduce the computer memory footprint of the *k*-mer count tables, the *k*-mer strings were recoded as integers, split into separate lexicographical groups according to the leading nucleotides (i.e. *k*-mers beginning with *AAA*, *AAC*, etc. were assigned to different groups), and, within each set, sorted lexicographically again. In total, we recorded the frequency of 1,734,834,987 *k*-mers across 71 samples. The resulting count tables were filtered to retain *k*-mers showing variation in the population: we discarded *k*-mers present in fewer than 3 samples, or absent in fewer than 3 samples. These filters removed 967,274,879 *k*-mers, leaving 767,560,108 *k*-mers for further analysis.

To test whether the frequencies of any *k*-mers were associated with pirimiphos-methyl resistance, normalised *k*-mer frequencies were obtained by dividing the *k*-mer counts by the total number of variant *k*-mers in each sample. We calculated the Spearman’s rank correlation of each *k*-mer frequency with the resistance phenotype for each of the 767,560,108 *k*-mers (*cor*.*test* function in the *R stats* library [82]), correcting for multiple testing using local estimation of false discovery rates (*fdrtool* function in *fdrtool* library [79]), and using a significance threshold of *FDR* < 0.001.

Given that multiple *k*-mers can overlap a single mutation, it was likely that many of the *k*-mers identified as significant were overlapping. We therefore took the *k*-mers that showed a significant association with pirimiphos-methyl resistance and assembled them by joining any *k*-mers that overlapped perfectly over at least 10 bp. The resulting assembled *k*-mers were aligned against the *A*. *gambiae* reference genome using *bwa mem* version 0.7.12-r1034 (parameters: *-T 0*) [51]. The mapping coordinates and sequences of the significant assembled *k*-mers are available in S8 Data.

A minority of assembled *k*-mers aligned in regions other than the *Ace1* duplication. To determine whether this was due to mis-alignment, we correlated the frequency of these assembled *k*-mers in each sample against the *Ace1* copy number. Frequency for each assembled *k*-mer was calculated as the mean normalised frequency of all the *k*-mers that were used in its assembly. Because these *k*-mers and the *Ace1* copy number share the property of being correlated with pirimiphos-methyl resistance phenotype, they are statistically likely to have correlated frequencies even if physically unlinked. To control for this, the residuals of *k*-mer frequency and *Ace1* copy number were calculated within each of the resistant and susceptible groups of samples. Pearson’s correlation was then calculated on these residuals for each of the assembled *k*-mers (available in S8C Data).

Scripts to reproduce the *k*-mer analysis steps described above (counting, filtering, significance testing, assembly, mapping, and *Ace1* correlation analysis) are available on Github (see Availability of data and materials).

### Alignment and phylogenetic analysis of ACE proteins in animals

To obtain a candidate list of homologs of the *Ace1* gene, we retrieved all genes belonging to the orthogroup 339133at33208 from OrthoDB [83] (682 genes in total). We aligned these candidates to a curated database of predicted proteomes from 89 complete animal genomes (listed in S2 Data, including data sources) using Diamond 0.9.22.123 [84], and retained all alignments with an identity >95% to any of the candidate queries (130 genes). Next, we performed a multi-sequence alignment with *MAFFT* 7.310 (1,000 rounds of iterative refinement, L-INS-i algorithm) [85] of these 130 genes, together with the truncated sequence of the Pacific electric ray *Torpedo californica* protein used for its crystal structure analysis (PDB accession number: 1W75) and the full-sequence of the ortholog from its close relative *T*. *marmorata* (Uniprot accession number: P07692). This alignment (n = 132 genes) was trimmed position-wise using *trimAL* 1.4 [86] (*automated1* mode), and 277 conserved columns were retained. A maximum-likelihood phylogenetic analysis of the trimmed alignment was then performed using *IQ-TREE* 1.6.10 [70], using a LG substitution matrix [87] with four Γ categories and accounting for invariant sites (*LG+I+G4* model). *IQ-TREE* was run for 469 iterations until convergence was attained (correlation coefficient ≥ 0.99). Node statistical supports were calculated using the UF bootstrap procedure (1,000 replicates) [72,73]. Alignment visualizations (S1 Data) were obtained from Geneious 11.1.4 [88]. Complete alignments are available as S2 Data.

## Supporting information

S1 Data Homology of *Ace1* mutations. **A)** Alignment of ACE homologs (protein sequences) in selected species (*A*. *gambiae*, *Culex quinquefasciatus*, *Aedes aegypti*, *Homo sapiens*, and *Torpedo californica*), used to determine the homology of non-synonymous mutations in this gene (*A65S* and *G280S* are highlighted). **B)** Alignment of ACE protein homologs in 20 culicine species, focusing on the vicinity of codon 280 (highlighted). **C)** Maximum-Likelihood phylogenetic analysis of ACE homologs from 89 animals (listed in S2 Data, including data sources and accession numbers).
(PDF)

S2 Data Alignments of ACE homologs. **A)** Peptide coordinates of *A*. *gambiae Ace1* codon 280 in orthologs of *Ace1* from 89 animal genomes. **B)** Alignment of *Ace1* homologs from 89 animal genomes. **C)** Data sources, accession numbers, species abbreviation and taxonomy of the 89 animal genomes used in the analysis of ACE homology.
(XLSX)

S3 Data *Ace1* mutations. Coordinates of non-synonymous mutations in *Ace1* and frequencies in each population of the *Anopheles gambiae* 1000 Genomes Phase 2 dataset. Species codes: *A*. *gambiae*, gam, *A*. *coluzzii*, col. Population codes: Angola, AOcol; Burkina-Faso, BFcol and BFgam; Côte d’Ivoire, CIcol; Cameroon, CMgam; Mayotte, FRgam; Gabon, GAgam; Ghana, GHcol and GHgam; The Gambia, GM; Guinea, GNcol and GNgam; Equatorial Guinea, GQgam; Guinea-Bissau, GW; Kenya, KE; Uganda, UGgam.
(XLSX)

S4 Data Samples from *Ag1000G* Phase 2. List of samples from the *Anopheles gambiae* 1000 Genomes Phase 2 dataset with accession numbers, sample metadata (population and region of origin, collection date, species, sex), and summary of the main *Ace1* mutations in each sample (genotypes in the *G280S* mutation, number of reads supporting *280G* and *280S* alleles, number of CNVs). CNV coordinates and copy number from Lucas *et al*. (2019).
(XLSB)

S5 Data Genes in *Ace1* duplication. List of genes in the *Ace1* duplication region, with genomic coordinates along the 2R chromosomal arm.
(XLSX)

S6 Data Phenotype-genotype association analyses. **A)** Genotypes of *G280S* and *A65S* mutations in *A*. *coluzzii* samples from Côte d’Ivoire (*Anopheles gambiae* 1000 Genomes Phase 2), and resistance phenotype to pirimiphos-methyl. **B)** Phenotype-genotype association tests for the 71 Ivorian *A*. *coluzzii* samples. Includes summaries of single-variable GLM models for the following variables: *280S* presence (including a subset of samples with only one *280S* allele), *65S*, CNV and number of *280S* alleles. Also includes the minimal model obtained using stepwise reduction of a starting multi-variable model (BIC criterion). Model significance is measured with ANOVA comparison with null model (no variables) and a χ^2^ test. **C)** *G280S* genotypes and pirimiphos-methyl resistance phenotype for 1080 mosquitoes from West African populations of *A*. *gambiae* and *A*. *coluzzii* collected from six locations. For each sample, we also report collection dates, geographical locations, and details of the species identification, genotyping, and phenotyping (concentrations, exposure time). **D)** Phenotype-genotype association tests for *G280S* alleles in nine West African populations. Includes summaries of GLM models for each population. Model significance is measured with ANOVA comparison with null model (no variables) and a χ^2^ test. **E)** Number of *Ace1* copies and pirimiphos-methyl resistance phenotypes for 167 mosquitoes from the same West African populations. **F)** Phenotype-genotype association tests for CNV mutations (*Ace1* copies [CNV] and *280S-*to-*280G* allele ratio [ratio_FAM_HEX]) in West African populations. Includes summaries of GLM models for each population and each variable separately, and a minimal GLM obtained using the BIC criterion from an initial model with both variables. Model significance is measured with ANOVA comparison with null model (no variables) and a χ^2^ test. **G)** Protocols for species identification and *Ace1* mutation genotyping, including primer sequences.
(XLSX)

S7 Data Genetic differentiation in Ivorian *A*. *coluzzii*. **A)** Genetic differentiation statistics (Hudson’s *F*_*ST*_ and *PBS*) between resistant and susceptible *A*. *coluzzii* from Côte d’Ivoire, in windows of 10,000 variants along the genome. *PBS* values are calculated using Angolan *A*. *coluzzii* as outgroup, and we also report a *Z*-score and *p*-value derived from a two-sided normal distribution. **B)** Genes overlapping the regions of high *PBS* (*FDR* < 0.001 and standardised *PBS* > 0).
(XLSX)

S8 Data *k*-mer analysis in Ivorian *A*. *coluzzii*. **A)** Alignment coordinates of *k*-mers that are significantly associated with pirimiphos-methyl resistance in Ivorian *A*. *coluzzii* samples from the *Anopheles gambiae* 1000 Genomes Phase 2. For each *k*-mer, we report the alignment sequence and coordinates, and whether it overlaps the *Ace1* gene or the *Ace1* duplication. This table includes both primary and secondary alignments as reported by *bwa mem*, as well as non-aligned *k*-mers (chromosome “NA”). **B)** Correlation of alignment frequencies and *Ace1* copy number in each sample, for each of the 409 *k-*mers that mapped to chromosomal arms 2R, 2L, 3R, 3L or X. We report Pearson’s correlation coefficients (*r*) and *p-*values. **C)** Sequences of the 446 assembled *k*-mers that are significantly associated with pirimiphos-methyl resistance. **D)** Frequencies of the 9,603 *k-*mers (before assembly) in each of the Côte d’Ivoire *A*. *coluzzii* samples (*Anopheles gambiae* 1000 Genomes Phase 2, n = 71).
(XLSX)

S9 Data Identification of tagging variants for *Ace1 280S*. **A)** Density of phased variants in the *Ace1* duplication region, from *Anopheles gambiae* 1000 Genomes Phase 2. **B)** Density of phased variants within the *Ace1* duplication, focusing on the *Ace1* gene body. **C)** Linkage disequilibrium between the *G280S* alleles and nearby phased variants (Huff and Roger’s *r*), highlighting the coordinates of nearby variants tightly linked to *280S* alleles that could be used as tagging variants for *280S* (in green), or variants that were later discarded by other filtering steps (in grey), and their frequencies in Côte d’Ivoire *A*. *coluzzii*. **D)** Haplotype score values in the vicinity of the *Ace1* locus, calculated from the Phase 2 1000 Genomes cohort. Variants within low-score regions can be used to tag *280S* (in green), variants within peaks were discarded (grey). **E)** Distribution of haplotype score values for variants within the regions used to build haplotype networks (green, tagging variant ± 300 bp) and in the *Ace1* gene body (magenta). Distributions are shown as cumulative frequency distributions and boxplots (box lines represent the first, second and third quartiles). **F)** Distribution of the sequencing coverage of each sample with *Ace1* duplications, for the genomic window containing each tagging variant (green) and *Ace1* (magenta). A normalised coverage of two implies diploidy. Tagging variants are located in regions of lower sequencing coverage than *Ace1*, even though they all fall within the duplicated region.
(PDF)

S10 Data Haplotype networks around the *Ace1* tagging variants. Minimum spanning tree networks built from phased variants located around each of the three tagging variants (panels A to C). Each node in the network is colored according to its population composition (left panels) or linkage to the *280S* or *wt* alleles in *Ace1* (right panels).
(PDF)

S11 Data Signals of selection in the *Ace1* duplication. Garud *H* statistics and haplotype diversity in *280S*-linked and *wt-*linked haplotypes in the genomic window around the *Ace1* duplication breakpoints, calculated for each of the main haplotype clusters defined around each of the three tagging variants (panels A to C; haplotype clusters from S10). For each tagging variant and duplication breakpoint (upstream/downstream), we report the average value of each statistic and standard errors from sample jack-knifing.
(PDF)

S12 Data Extended haplotype homozygosity in the *Ace1* duplication. Extended haplotype homozygosity (*EHH*) of *280S*-linked and *wt-*linked haplotypes in the genomic window around the *Ace1* duplication breakpoints, calculated for each of the main haplotype clusters defined around each of the four tagging variants (panels A to D; haplotype clusters from S10). For each tagging variant and duplication breakpoint (upstream/downstream), we report the area under the *EHH* curve (*a*) and the distance around the duplication breakpoint where *EHH*>0.05 and *EHH*>0.95.
(PDF)

S13 Data Introgression of the *Ace1* duplication. Patterson *D* statistics to test introgression of the *Ace1* duplication region between various populations of *A*. *coluzzii* (populations A/B), *A*. *gambiae* (populations C) and multiple outgroup species (population O). Specifically, we use *A*. *coluzzii* from Côte d’Ivoire (CIcol), Ghana (GHcol) and Burkina-Faso (BFcol) with and without duplications (labelled as TRUE and FALSE respectively) as populations A; *A*. *coluzzii* from Angola as population B (always labelled as FALSE); and various *A*. *gambiae* populations C from Ghana (GHgam), Burkina-Faso (BFgam), Guinea (GNgam), Gabon (GAgam) and Cameroon (CMgam) with and without duplications (TRUE and FALSE labels, respectively); and four different outgroup species (panels A to D: *A*. *arabiensis*, *A*. *melas*, *A*. *merus* and *A*. *quadriannulatus*). For each comparison, we report the average *D* statistic from the *Ace1* duplicated region with standard errors, and *Z-*scores and *p*-values derived from standardised *D* values (unit variance).
(PDF)

S14 Data Haplotype alignments. **A)** Alignment of phased variants from within the *Ace1* duplication region (2R:3,436,800–3,639,600), using 345 samples from West African populations (*Anopheles gambiae* 1000 Genomes) with *Ace1* duplications (Guinea *A*. *gambiae*, Côte d’Ivoire *A*. *coluzzii*, Ghana *A*. *gambiae* and *A*. *coluzzii*, Burkina Faso *A*. *gambiae* and *A*. *coluzzii*). **B)** Id., from a region downstream of the duplication (5 kbp starting at 3,639,600). **C)** Id., from a region upstream of the duplication (50 kbp starting at 3,436,800–1 Mb). **D)** Id., from a region downstream of the duplication (50 kbp starting at 3,639,600 + 1 Mb).
(XLSX)

S15 Data Haplotype phylogenies. Maximum-Likelihood phylogenetic analyses of haplotypes from within the *Ace1* duplication (A), downstream breakpoint (B), upstream (C) and downstream (D) regions. Trees are unrooted. Tips are color-coded according to duplication presence/absence and species. UF bootstrap supports indicated in each node.
(PDF)

S16 Data Signals of selection in differentiated genomic windows. **A)** Neighbour joining trees built from phased variants located within the *PBS* peaks identified between resistant and susceptible Ivorian *A*. *coluzzii* (1000 Genomes dataset). **B)** Profile of Garud’s *H*_*12*_ in these same regions.
(PDF)

S17 Data Sequencing coverage along the *Ace1* CNV. **A)** Sequence of the duplication breakpoints described by Assogba *et al*. (2016) with their coordinates in the *A*. *gambiae* genome assembly (AgamP4) and the corresponding genome window in the CNV database by Lucas *et al*. (2019). **B)** Normalised sequencing coverage along the *Ace1* duplication locus in each sample of the *Anopheles gambiae* 1000 Genomes dataset (grouped by population of origin). Detailed coverage of each sample available in S4B Data.
(PDF)

S18 Data **A)** Distance in allelic frequencies between *A*. *coluzzii* specimens with duplications and *wt A*. *coluzzii* and *A*. *gambiae*, calculated using the three-population branch statistic in windows of 5,000 variants along the genome. Includes estimated distance (*L*) from within the duplication region. **B)** Id., using *A*. *gambiae* specimens with duplications.
(PDF)
