# Supplementary material for: Resistance to pirimiphos-methyl in West African Anopheles is spreading via duplication and introgression of the Ace1 locus
Source: PLoS Genet. 2021 Jan 21;17(1):e1009253. doi: 10.1371/journal.pgen.1009253 (PMC7853456; doi:10.1371/journal.pgen.1009253)

Supplementary Material SM1

A) Alignment of *Ace1* orthologs: selected animal sequences

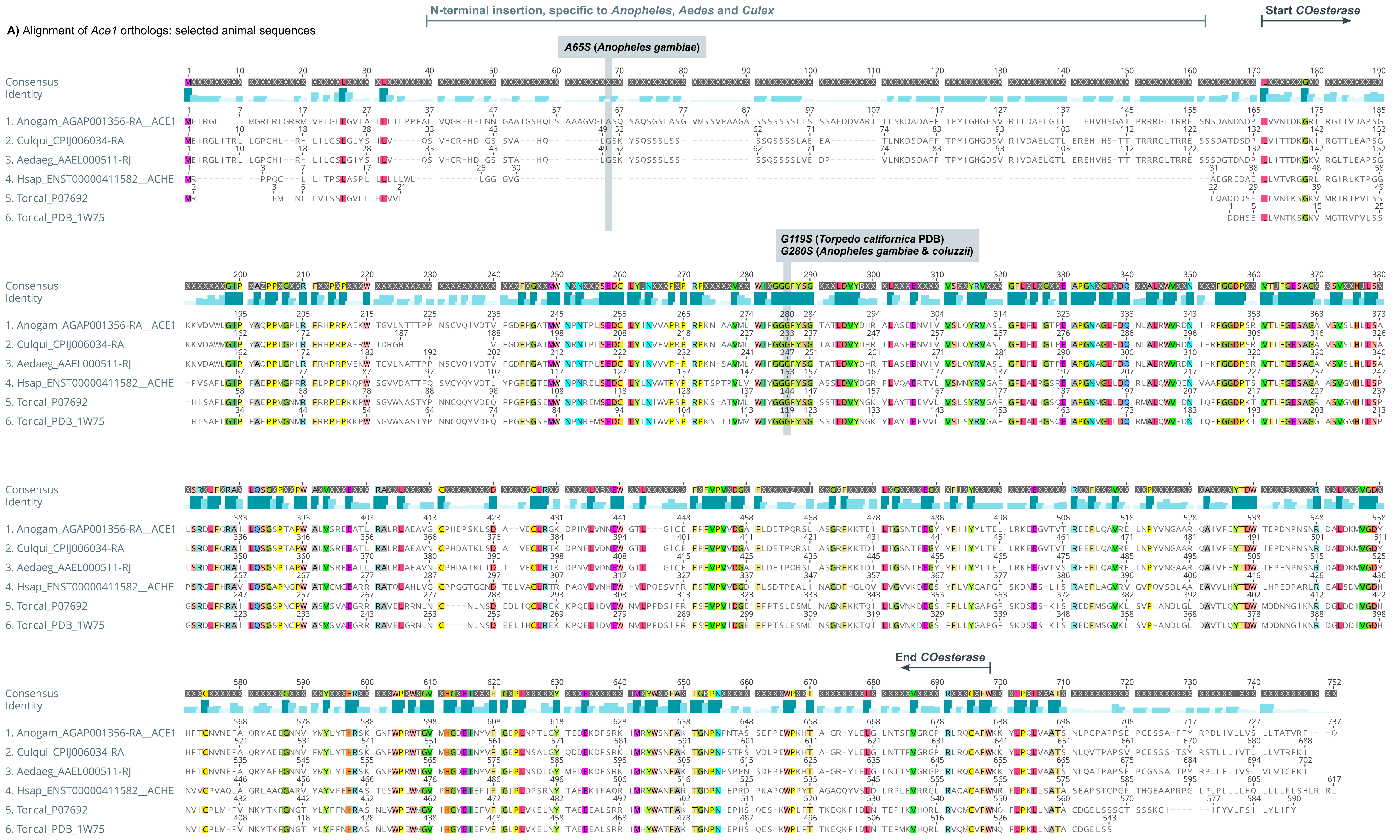

B) Alignment of *Ace1* orthologs: *Anopheles*, *Culex* and *Aedes*

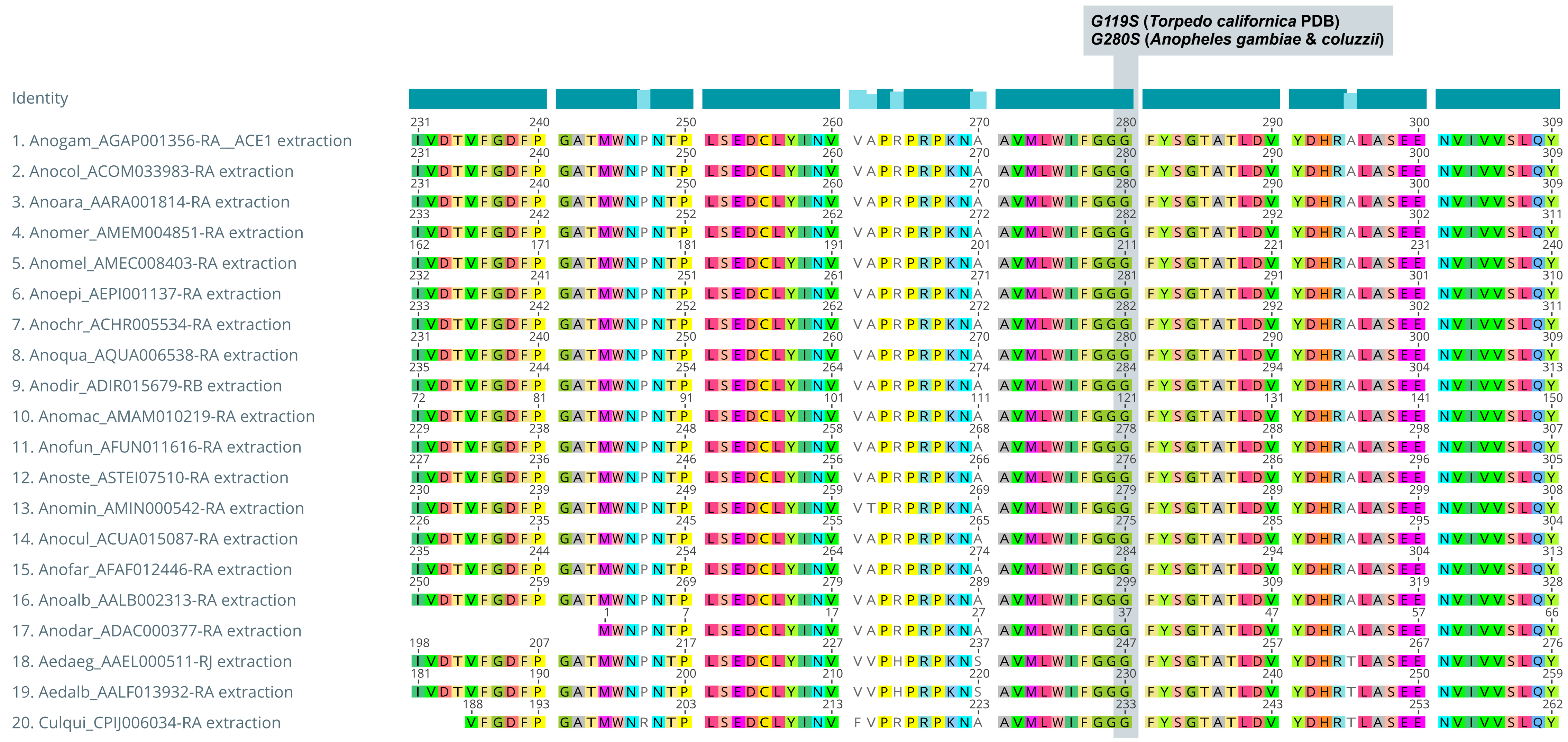

C) Phylogenetic analysis Ace homologs

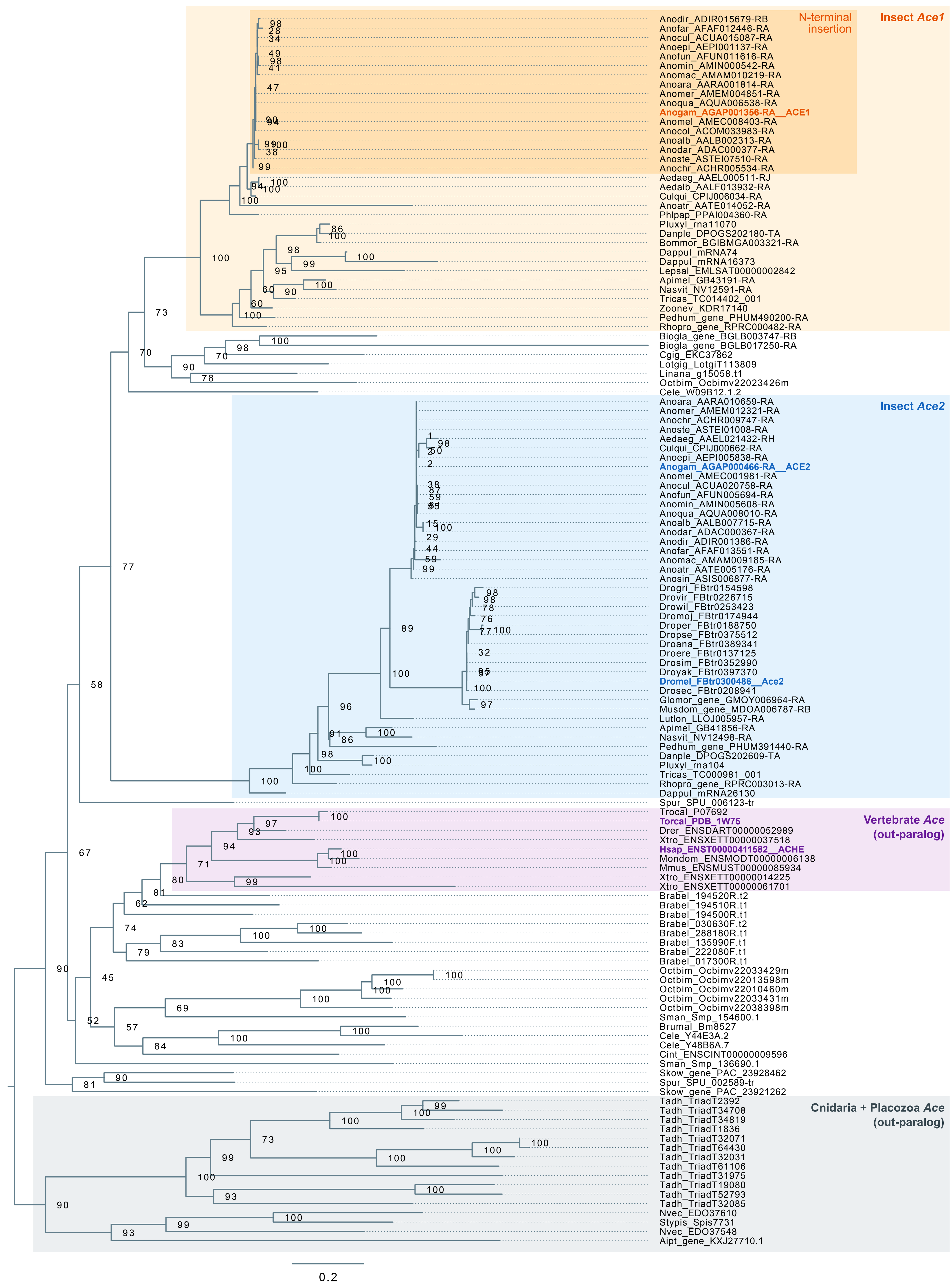

Supplement: S1 Data — A) Alignment of ACE homologs (protein sequences) in selected species (A. gambiae, Culex quinquefasciatus, Aedes aegypti, Homo sapiens, and Torpedo californica), used to determine the homology of non-synonymous mutations in this gene (A65S and G280S are highlighted). B) Alignment of ACE protein homologs in 20 culicine species, focusing on the vicinity of codon 280 (highlighted). C) Maximum-Likelihood phylogenetic analysis of ACE homologs from 89 animals (listed in S2 Data, including data sources and accession numbers). (PDF) [file pgen.1009253.s001.pdf]
