# Supplementary material for: Resistance to pirimiphos-methyl in West African Anopheles is spreading via duplication and introgression of the Ace1 locus
Source: PLoS Genet. 2021 Jan 21;17(1):e1009253. doi: 10.1371/journal.pgen.1009253 (PMC7853456; doi:10.1371/journal.pgen.1009253)

Supplementary Material SM9

A) Density of phased variants around *Ace1* duplication region

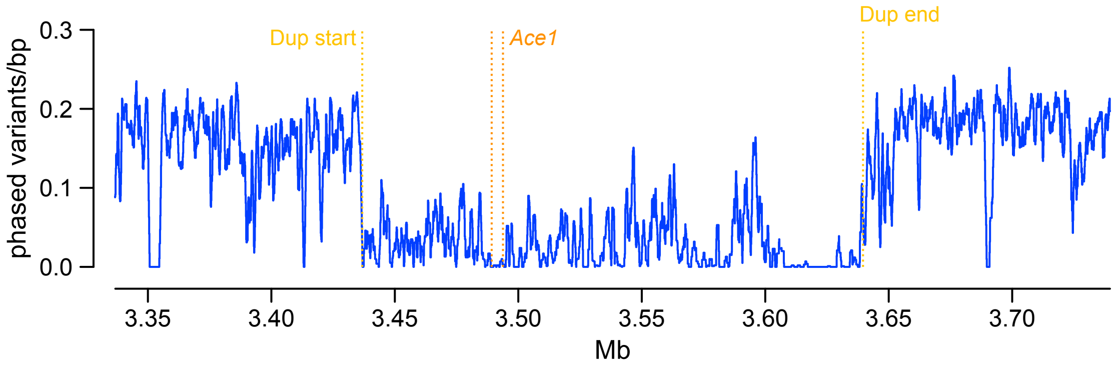

B) Density of phased variants within *Ace1* duplication region

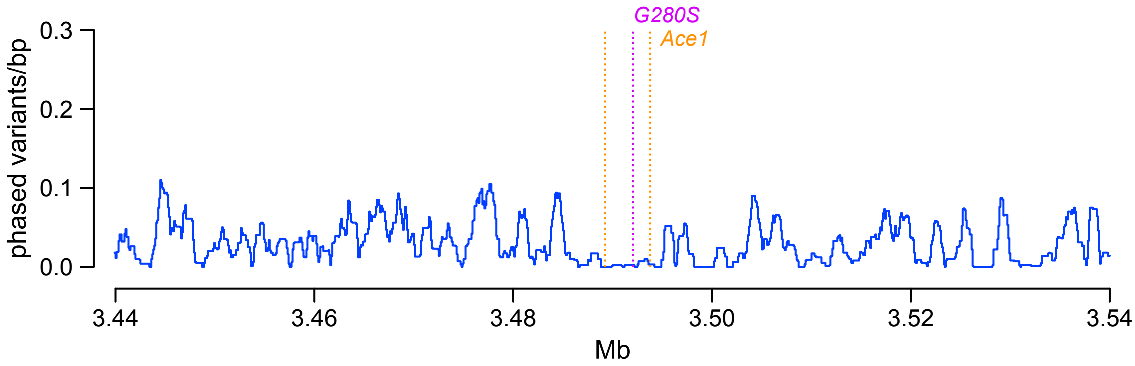

C) Linkage disequilibrium between *GG280S* and nearby phased variants

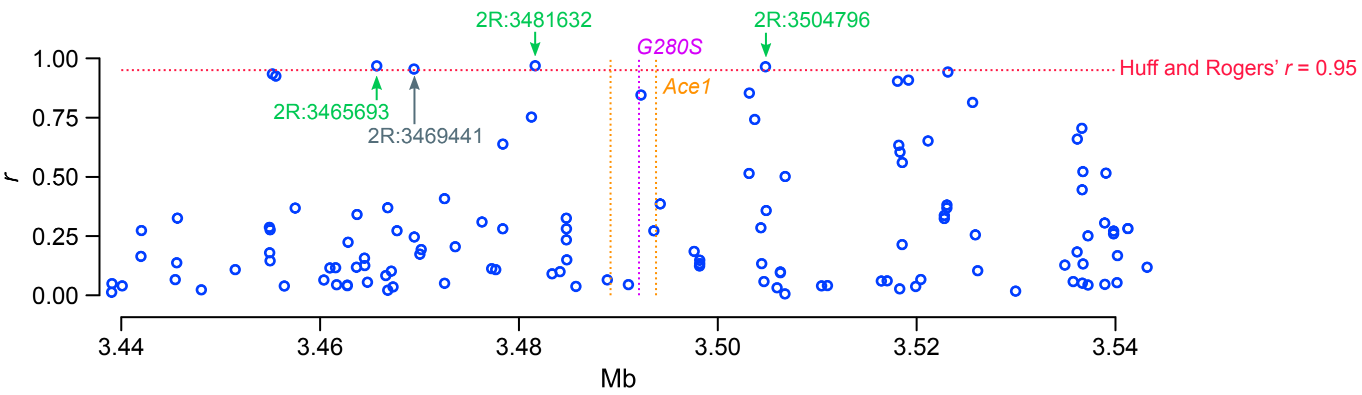

chrom	pos	LD with G280S	Freq CIcol	Phased?
2R	3444618	0.97264516	0.4366	0
2R	3445910	0.9504605	0.4366	0
2R	3447556	0.96361303	0.4366	0
2R	3456166	0.9675373	0.4366	0
2R	3456937	0.97637683	0.4366	0
2R	3459582	0.9591772	0.4366	0
2R	3464705	0.96810204	0.4366	0
**2R**	**3465693**	**0.96810204**	**0.4366**	**1** ***
2R	3466268	0.9719312	0.4366	0
**2R**	**3469441**	**0.95479333**	**0.4366**	**1**
2R	3469672	0.96308875	0.4366	0
2R	3475119	0.9658597	0.4366	0
2R	3477809	0.98811066	0.4366	0
2R	3478595	1.0	0.4366	0
2R	3479474	0.98421353	0.4366	0
2R	3480330	1.0	0.4366	0
2R	3480406	0.98421353	0.4366	0
2**R**	**3481632**	**0.96864766**	**0.4295**	**1** ***
2R	3482092	0.9716037	0.4154	0
2R	3483376	0.99236995	0.4366	0
2R	3483539	1.0	0.4366	0
2R	3486607	0.9959919	0.4366	0
2R	3487859	1.0	0.4366	0
2R	3496635	0.98388374	0.4366	0
2R	3498622	0.96866876	0.4366	0
2R	3498740	0.97213244	0.4366	0
**2R**	**3504796**	**0.96453327**	**0.4436**	**1** ***
2R	3507961	0.97605	0.4507	0

D) Profile of haplotype scores around *Ace1*

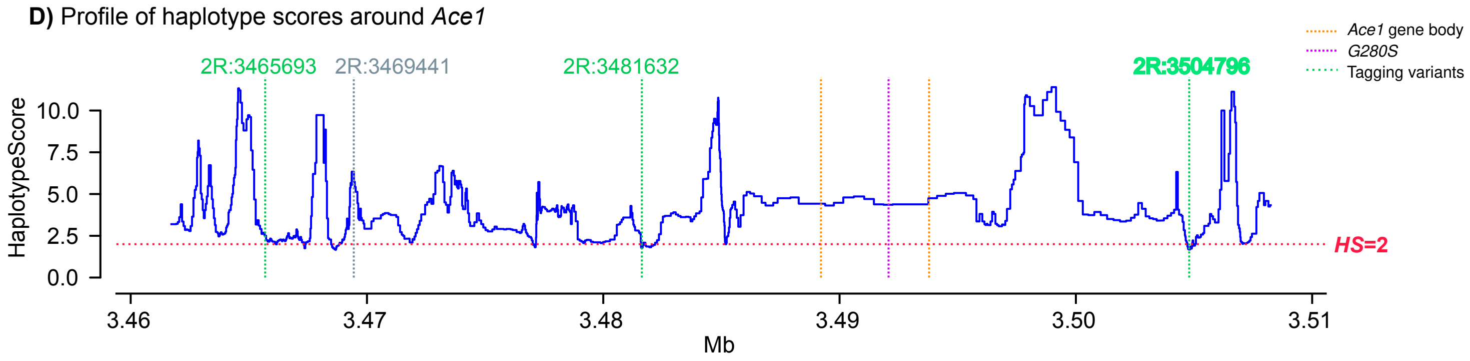

E) Haplotype score in *Ace1* and the tagging variants (+/-300bp)

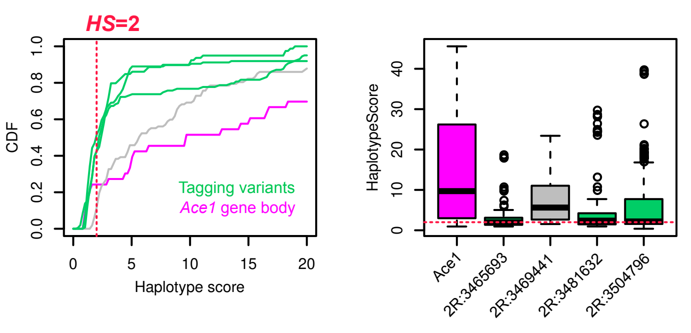

F) Sequencing depth in *Ace1* and the tagging variants (across samples)

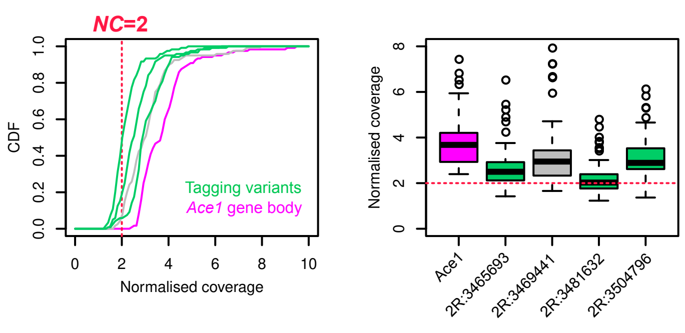

Supplement: S9 Data — A) Density of phased variants in the Ace1 duplication region, from Anopheles gambiae 1000 Genomes Phase 2. B) Density of phased variants within the Ace1 duplication, focusing on the Ace1 gene body. C) Linkage disequilibrium between the G280S alleles and nearby phased variants (Huff and Roger’s r), highlighting the coordinates of nearby variants tightly linked to 280S alleles that could be used as tagging variants for 280S (in green), or variants that were later discarded by other filtering steps (in grey), and their frequencies in Côte d’Ivoire A. coluzzii. D) Haplotype score values in the vicinity of the Ace1 locus, calculated from the Phase 2 1000 Genomes cohort. Variants within low-score regions can be used to tag 280S (in green), variants within peaks were discarded (grey). E) Distribution of haplotype score values for variants within the regions used to build haplotype networks (green, tagging variant ± 300 bp) and in the Ace1 gene body (magenta). Distributions are shown as cumulative frequency distributions and boxplots (box lines represent the first, second and third quartiles). F) Distribution of the sequencing coverage of each sample with Ace1 duplications, for the genomic window containing each tagging variant (green) and Ace1 (magenta). A normalised coverage of two implies diploidy. Tagging variants are located in regions of lower sequencing coverage than Ace1, even though they all fall within the duplicated region. (PDF) [file pgen.1009253.s009.pdf]
