# Supplementary material for: Resistance to pirimiphos-methyl in West African Anopheles is spreading via duplication and introgression of the Ace1 locus
Source: PLoS Genet. 2021 Jan 21;17(1):e1009253. doi: 10.1371/journal.pgen.1009253 (PMC7853456; doi:10.1371/journal.pgen.1009253)
Supplement: S10 Data — Minimum spanning tree networks built from phased variants located around each of the three tagging variants (panels A to C). Each node in the network is colored according to its population composition (left panels) or linkage to the 280S or wt alleles in Ace1 (right panels). (PDF) [file pgen.1009253.s010.pdf]

Supplementary Material SM10

A) Haplotype networks 2R:3465693

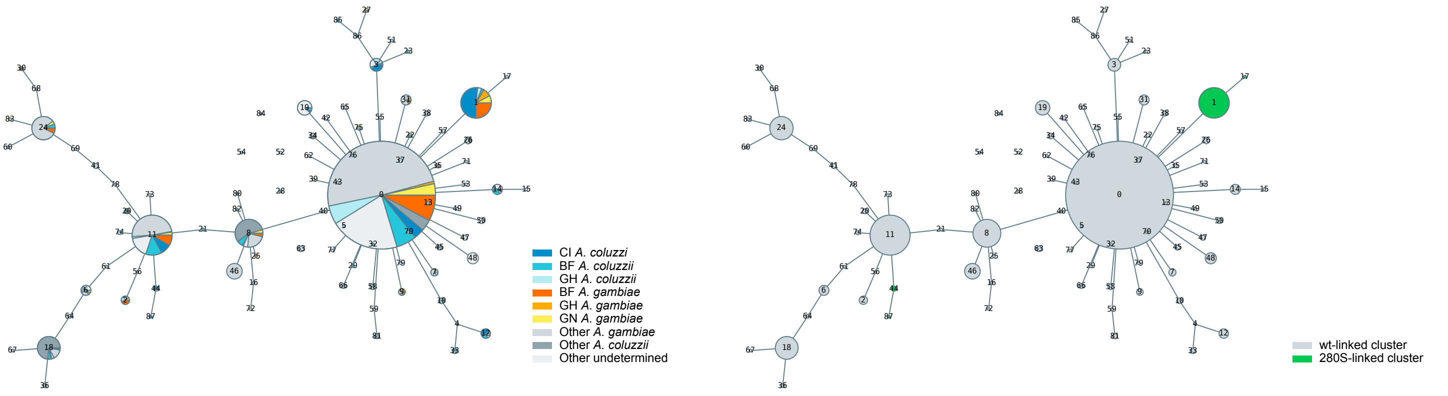

Ace1due 2R:3465693 . AGAP001355-AGA 2R:3465693 (3465693), allele 0
2284 haps clustered into 88 clusters with mst maxdist 1, from 67 phased vars located +/- 300 bp

B) Haplotype networks 2R:3481632

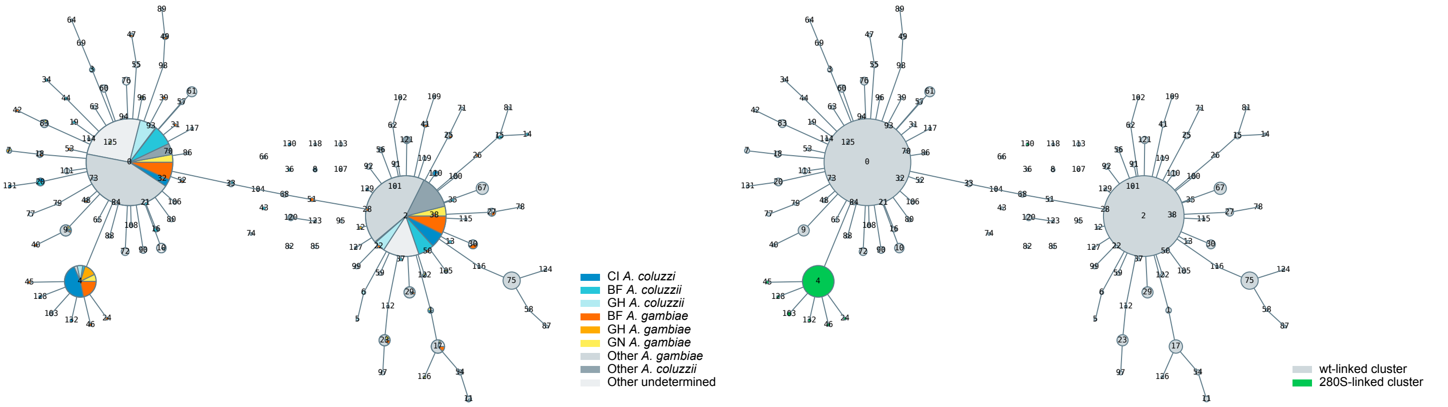

Ace1due 2R:3481632 . AGAP001356 2R:3481632 (3481632), allele 0
2284 haps clustered into 133 clusters with mst maxdist 1, from 104 phased vars located +/- 300 bp

C) Haplotype networks 2R:3504796

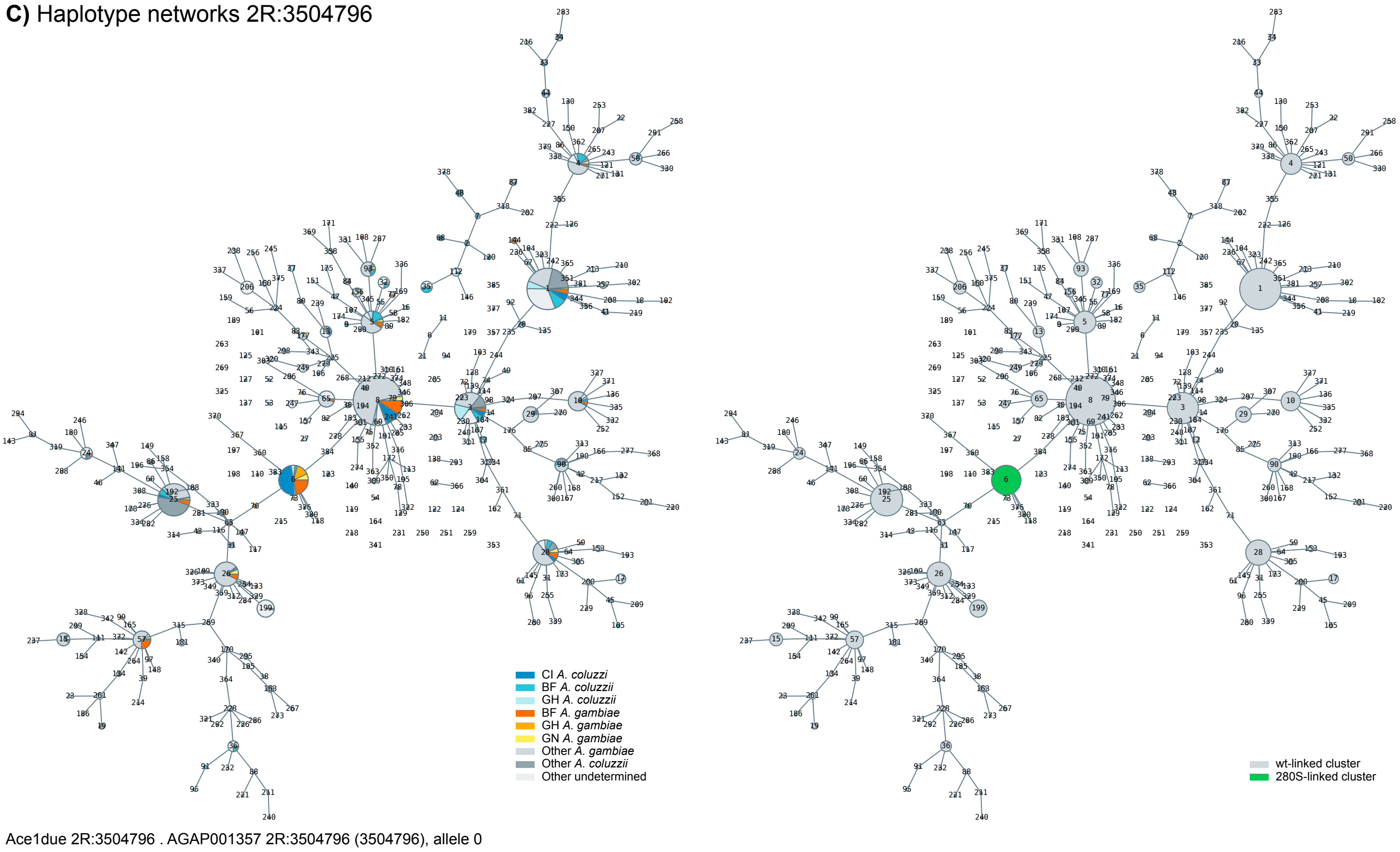

Ace1due 2R:3504796 . AGAP001357 2R:3504796 (3504796), allele 0
2284 haps clustered into 386 clusters with mst maxdist 1, from 164 phased vars located +/- 300 bp
