# Supplementary material for: Resistance to pirimiphos-methyl in West African Anopheles is spreading via duplication and introgression of the Ace1 locus
Source: PLoS Genet. 2021 Jan 21;17(1):e1009253. doi: 10.1371/journal.pgen.1009253 (PMC7853456; doi:10.1371/journal.pgen.1009253)
Supplement: S11 Data — Garud H statistics and haplotype diversity in 280S-linked and wt-linked haplotypes in the genomic window around the Ace1 duplication breakpoints, calculated for each of the main haplotype clusters defined around each of the three tagging variants (panels A to C; haplotype clusters from S10). For each tagging variant and duplication breakpoint (upstream/downstream), we report the average value of each statistic and standard errors from sample jack-knifing. (PDF) [file pgen.1009253.s011.pdf]

Supplementary Material SM11

A) Tagging variant 2R:3465693

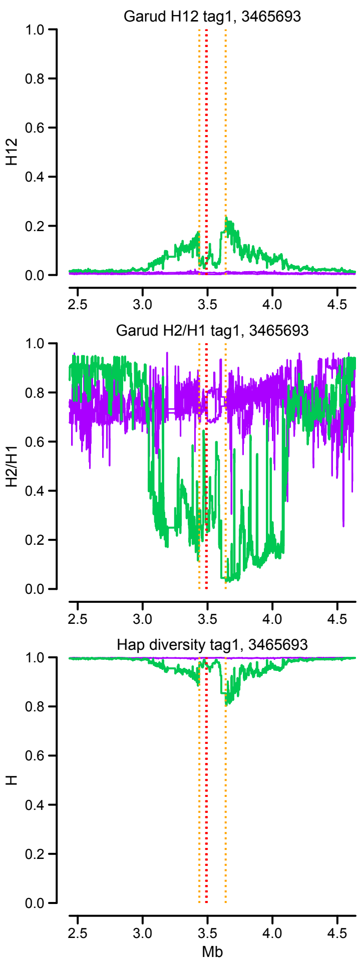


```
UPSTREAM

280S-linked cluster 1
h      = 0.853699 +/- 0.003219
H12    = 0.180403 +/- 0.003551
H2/H1 = 0.048027 +/- 0.001552

wt-linked cluster 0
h      = 0.997857 +/- 0.000008
H12    = 0.004081 +/- 0.000015
H2/H1 = 0.759491 +/- 0.001641

wt-linked cluster 11
h      = 0.997005 +/- 0.000091
H12    = 0.010303 +/- 0.000169
H2/H1 = 0.745234 +/- 0.009099


DOWNSTREAM

280S-linked cluster 1
h      = 0.915789 +/- 0.002090
H12    = 0.135794 +/- 0.002912
H2/H1 = 0.160537 +/- 0.005901

wt-linked cluster 0
h      = 0.997725 +/- 0.000007
H12    = 0.004034 +/- 0.000013
H2/H1 = 0.770208 +/- 0.001570

wt-linked cluster 11
h      = 0.996548 +/- 0.000095
H12    = 0.011212 +/- 0.000187
H2/H1 = 0.758875 +/- 0.008788
```

B) Tagging variant 2R:3481632

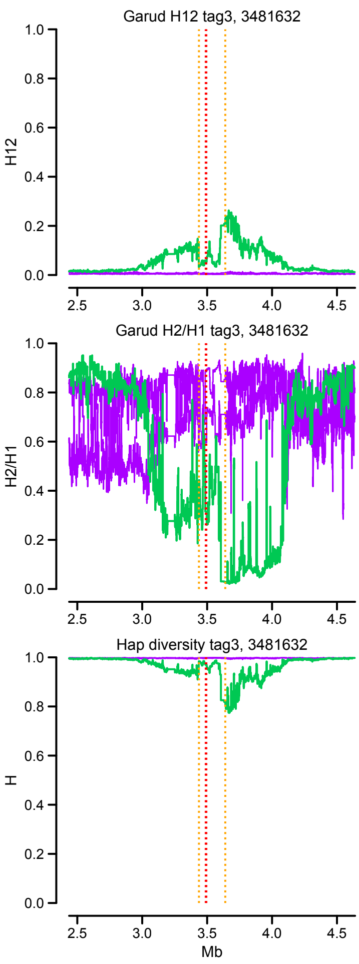


```
UPSTREAM

280S-linked cluster 4
h      = 0.818253 +/- 0.003414
H12    = 0.209079 +/- 0.003609
H2/H1 = 0.030246 +/- 0.000840

wt-linked cluster 0
h      = 0.998556 +/- 0.000008
H12    = 0.003125 +/- 0.000014
H2/H1 = 0.852989 +/- 0.001952

wt-linked cluster 2
h      = 0.995400 +/- 0.000026
H12    = 0.008875 +/- 0.000051
H2/H1 = 0.713415 +/- 0.002716


DOWNSTREAM

280S-linked cluster 4
h      = 0.949384 +/- 0.001251
H12    = 0.094103 +/- 0.002064
H2/H1 = 0.303158 +/- 0.009604

wt-linked cluster 0
h      = 0.997777 +/- 0.000013
H12    = 0.004349 +/- 0.000022
H2/H1 = 0.667241 +/- 0.002710

wt-linked cluster 2
h      = 0.995212 +/- 0.000026
H12    = 0.008356 +/- 0.000045
H2/H1 = 0.631817 +/- 0.002834
```

C) Tagging variant 2R:3504796

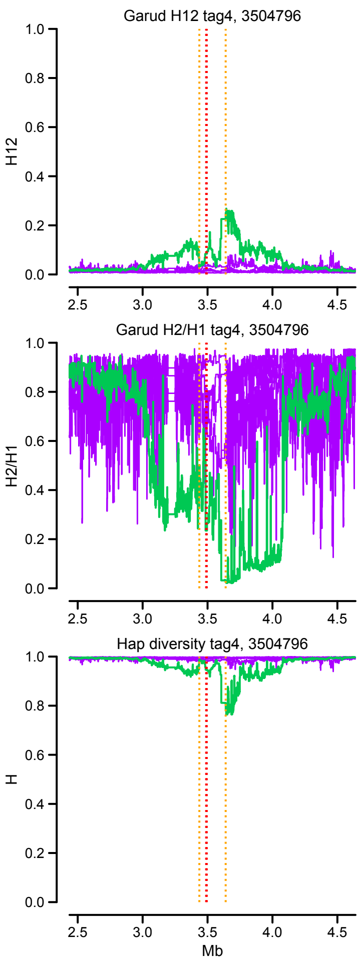


```
UPSTREAM

280S-linked cluster 6
h      = 0.811289 +/- 0.003744
H12    = 0.226071 +/- 0.004056
H2/H1 = 0.034603 +/- 0.001104

wt-linked cluster 1
h      = 0.998118 +/- 0.000034
H12    = 0.006827 +/- 0.000034
H2/H1 = 0.951386 +/- 0.000270

wt-linked cluster 25
h      = 0.986536 +/- 0.000385
H12    = 0.031173 +/- 0.000730
H2/H1 = 0.580588 +/- 0.012581

wt-linked cluster 3
h      = 0.996068 +/- 0.000138
H12    = 0.014405 +/- 0.000280
H2/H1 = 0.873700 +/- 0.001543

wt-linked cluster 8
h      = 0.989065 +/- 0.000126
H12    = 0.019955 +/- 0.000217
H2/H1 = 0.586079 +/- 0.005876


DOWNSTREAM

280S-linked cluster 6
h      = 0.970252 +/- 0.000886
H12    = 0.061395 +/- 0.001583
H2/H1 = 0.428783 +/- 0.013098

wt-linked cluster 1
h      = 0.997553 +/- 0.000050
H12    = 0.008096 +/- 0.000094
H2/H1 = 0.864587 +/- 0.005691

wt-linked cluster 25
h      = 0.991675 +/- 0.000202
H12    = 0.020459 +/- 0.000400
H2/H1 = 0.835920 +/- 0.002176

wt-linked cluster 3
h      = 0.996299 +/- 0.000131
H12    = 0.014176 +/- 0.000277
H2/H1 = 0.871137 +/- 0.001528

wt-linked cluster 8
h      = 0.989732 +/- 0.000122
H12    = 0.021415 +/- 0.000250
H2/H1 = 0.663398 +/- 0.005848
```
