# Supplementary figures and images for: Resistance to pirimiphos-methyl in West African Anopheles is spreading via duplication and introgression of the Ace1 locus
Source: PLoS Genet. 2021 Jan 21;17(1):e1009253. doi: 10.1371/journal.pgen.1009253 (PMC7853456; doi:10.1371/journal.pgen.1009253)

A) Tagging variant 2R:3465693

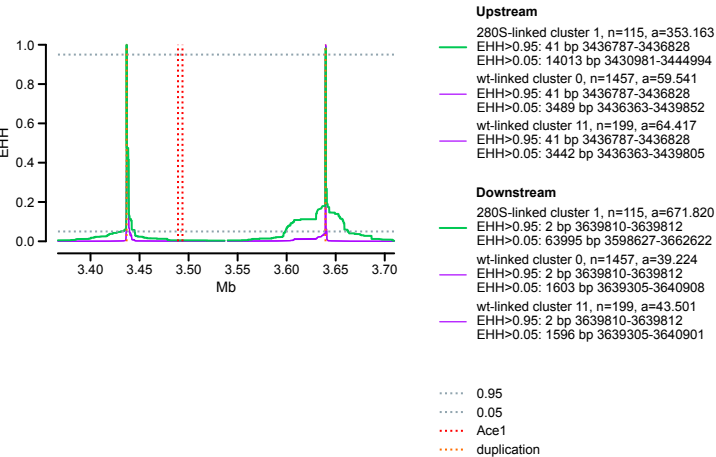

B) Tagging variant 2R:3469441

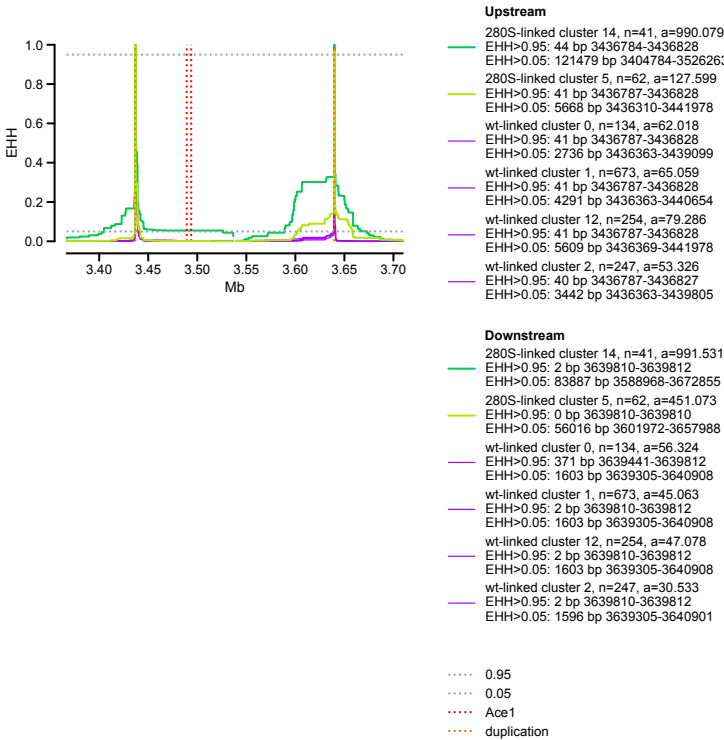

C) Tagging variant 2R:3481632

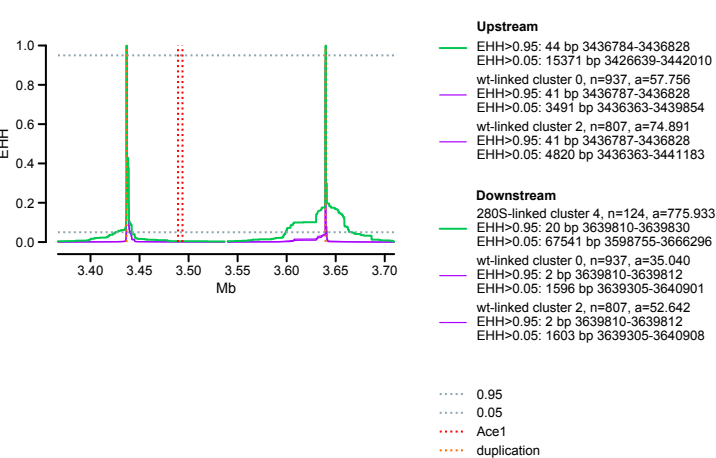

D) Tagging variant 2R:3504796

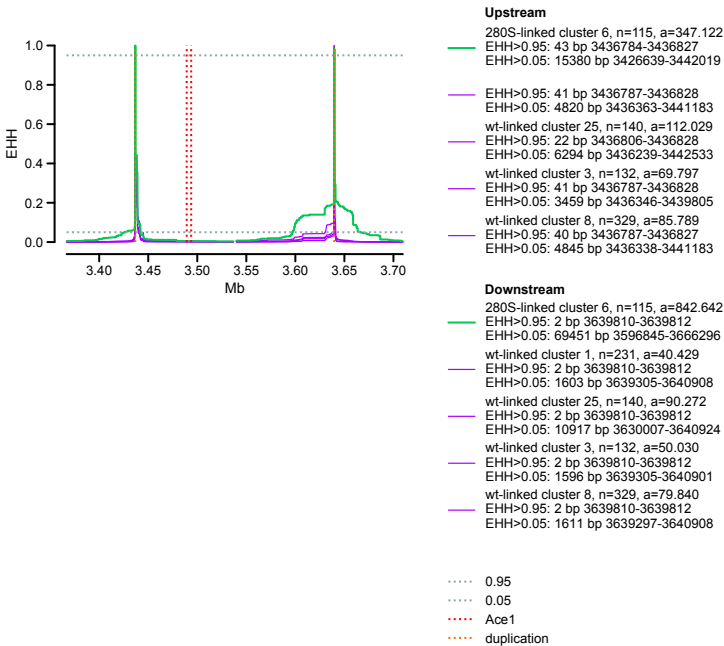

Supplement: S12 Data — Extended haplotype homozygosity (EHH) of 280S-linked and wt-linked haplotypes in the genomic window around the Ace1 duplication breakpoints, calculated for each of the main haplotype clusters defined around each of the four tagging variants (panels A to D; haplotype clusters from S10). For each tagging variant and duplication breakpoint (upstream/downstream), we report the area under the EHH curve (a) and the distance around the duplication breakpoint where EHH>0.05 and EHH>0.95. (PDF) [file pgen.1009253.s012.pdf]
