# Supplementary material for: Resistance to pirimiphos-methyl in West African Anopheles is spreading via duplication and introgression of the Ace1 locus
Source: PLoS Genet. 2021 Jan 21;17(1):e1009253. doi: 10.1371/journal.pgen.1009253 (PMC7853456; doi:10.1371/journal.pgen.1009253)

Supplementary Material SM13

A) Introgression in duplicated region, D=*A. arabiensis*

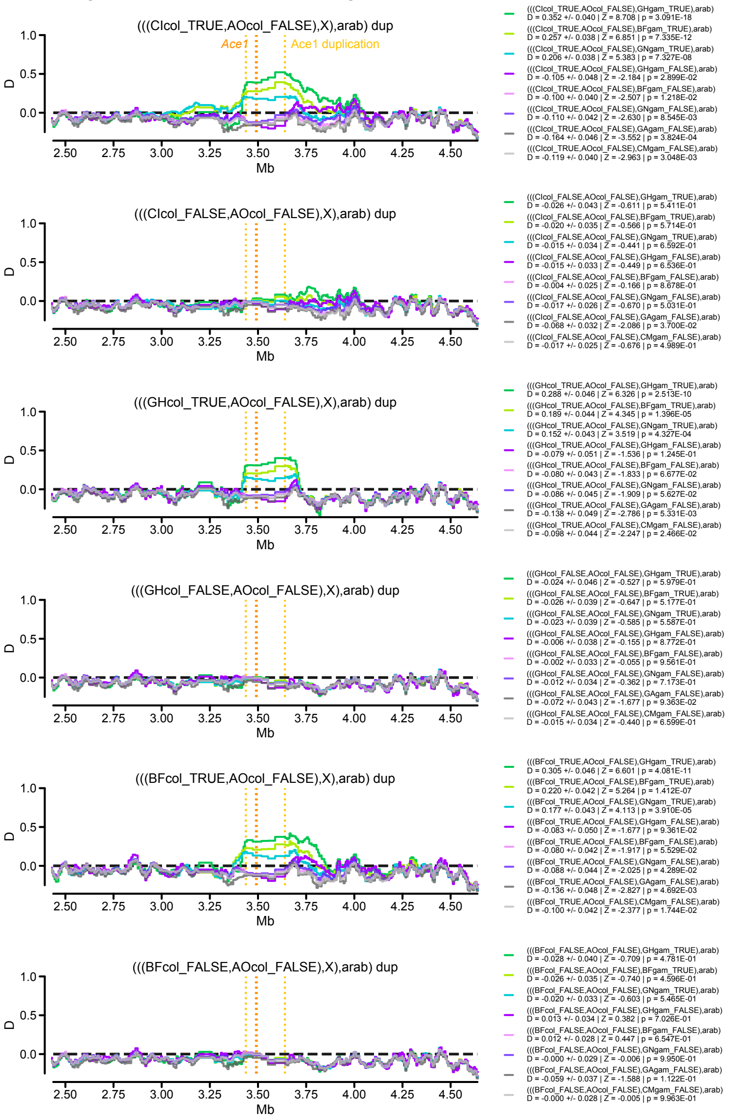

B) Introgression in duplicated region, D=*A. melas*

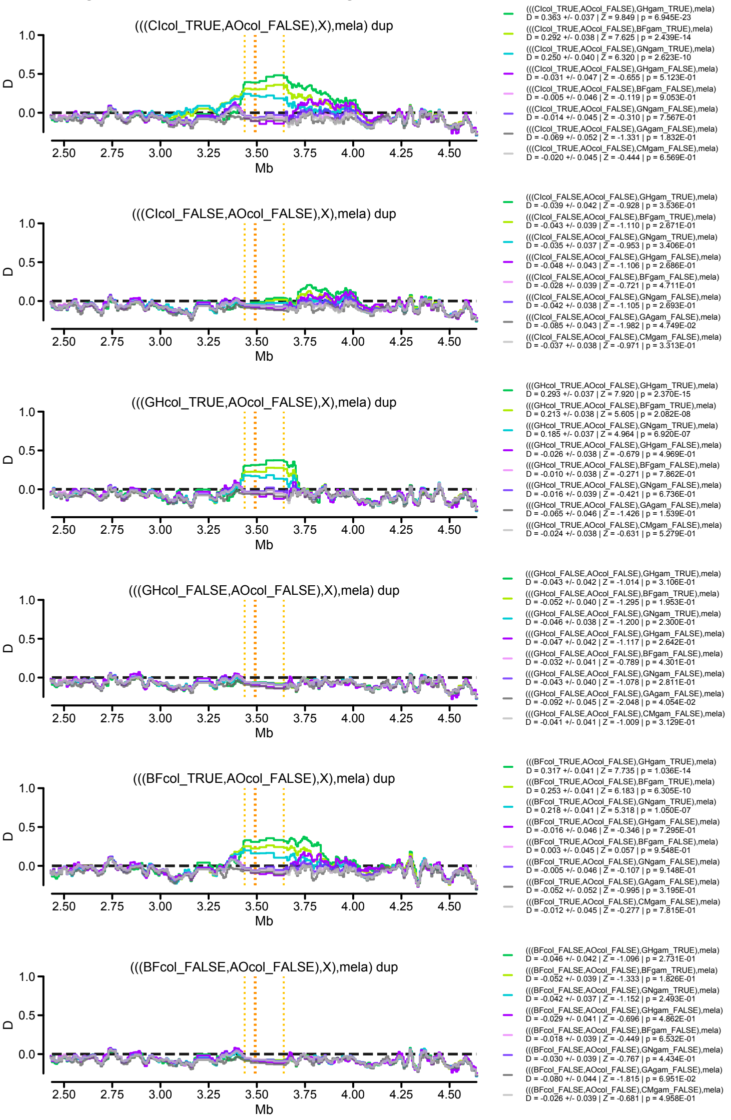

C) Introgression in duplicated region, D=*A. merus*

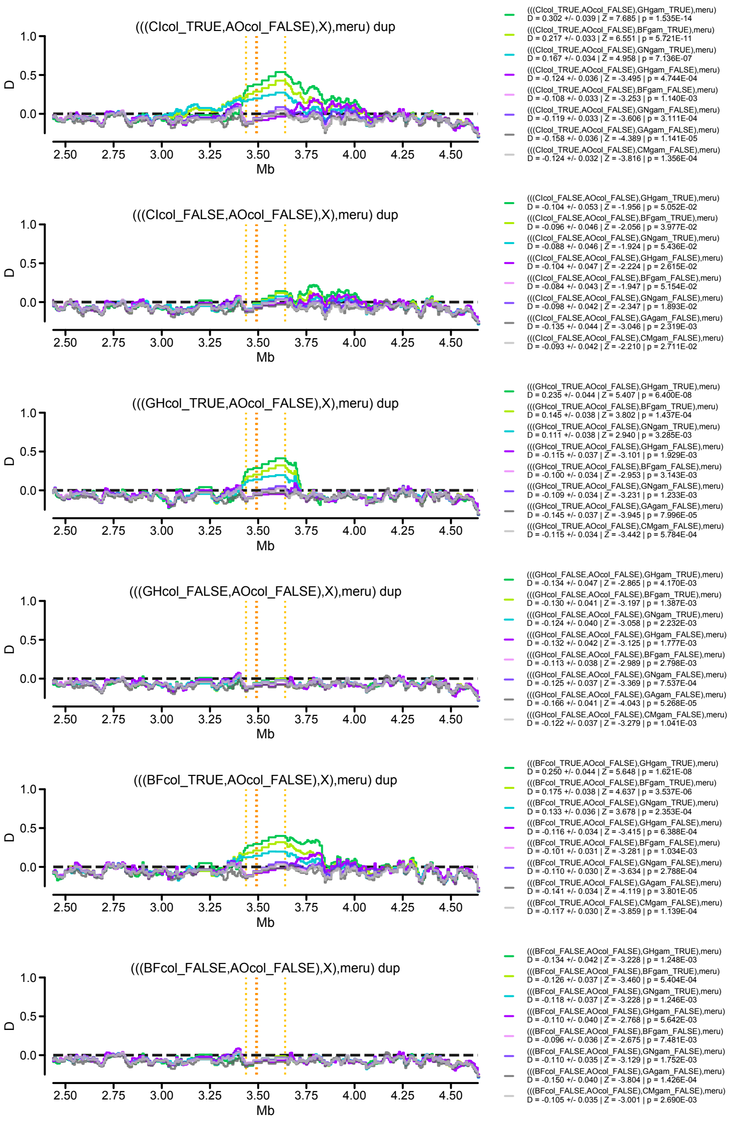

D) Introgression in duplicated region, D=*A. quadriannulatus*

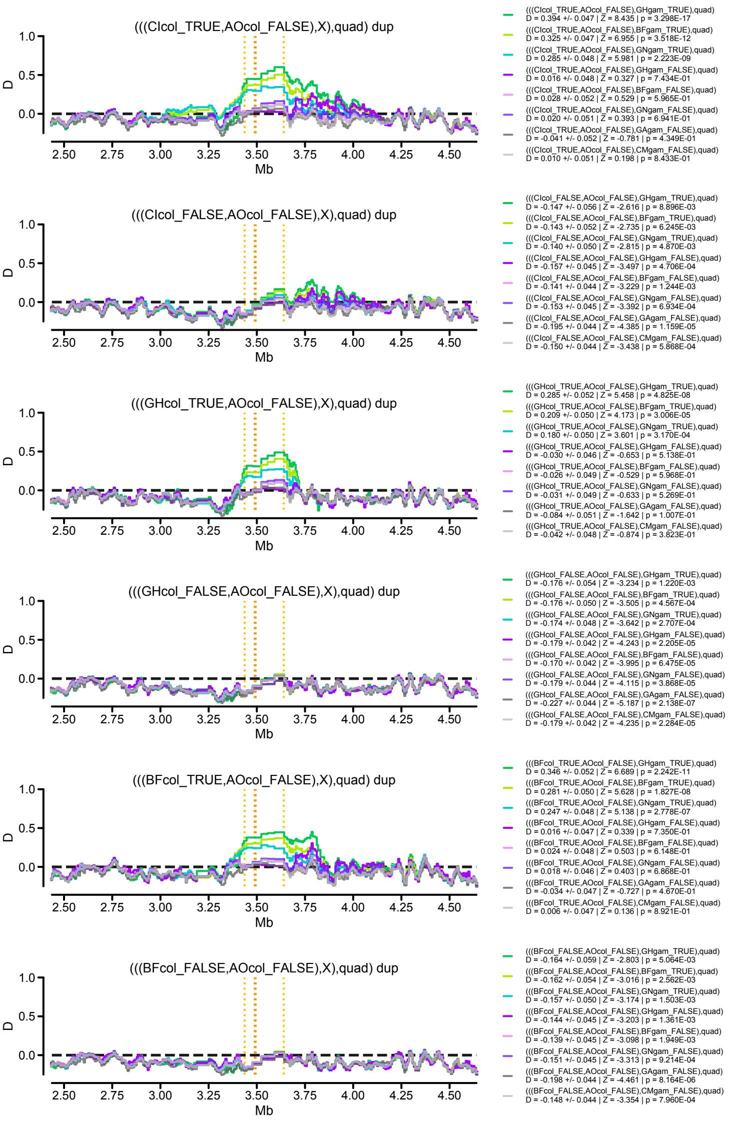

Supplement: S13 Data — Patterson D statistics to test introgression of the Ace1 duplication region between various populations of A. coluzzii (populations A/B), A. gambiae (populations C) and multiple outgroup species (population O). Specifically, we use A. coluzzii from Côte d’Ivoire (CIcol), Ghana (GHcol) and Burkina-Faso (BFcol) with and without duplications (labelled as TRUE and FALSE respectively) as populations A; A. coluzzii from Angola as population B (always labelled as FALSE); and various A. gambiae populations C from Ghana (GHgam), Burkina-Faso (BFgam), Guinea (GNgam), Gabon (GAgam) and Cameroon (CMgam) with and without duplications (TRUE and FALSE labels, respectively); and four different outgroup species (panels A to D: A. arabiensis, A. melas, A. merus and A. quadriannulatus). For each comparison, we report the average D statistic from the Ace1 duplicated region with standard errors, and Z-scores and p-values derived from standardised D values (unit variance). (PDF) [file pgen.1009253.s013.pdf]
