# Supplementary material for: Resistance to pirimiphos-methyl in West African Anopheles is spreading via duplication and introgression of the Ace1 locus
Source: PLoS Genet. 2021 Jan 21;17(1):e1009253. doi: 10.1371/journal.pgen.1009253 (PMC7853456; doi:10.1371/journal.pgen.1009253)

Supplementary Material SM15

A. coluzzii, no duplication
A. coluzzii, duplication
A. gambiae, no duplication
A. gambiae, duplication

A) Duplication

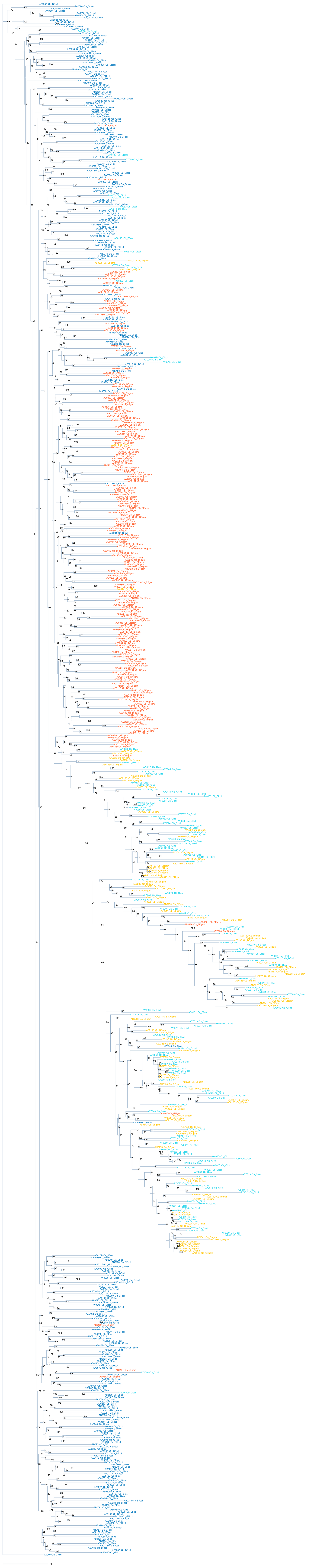

Supplementary Material SM15

B) Downstream of the duplication breakpoint

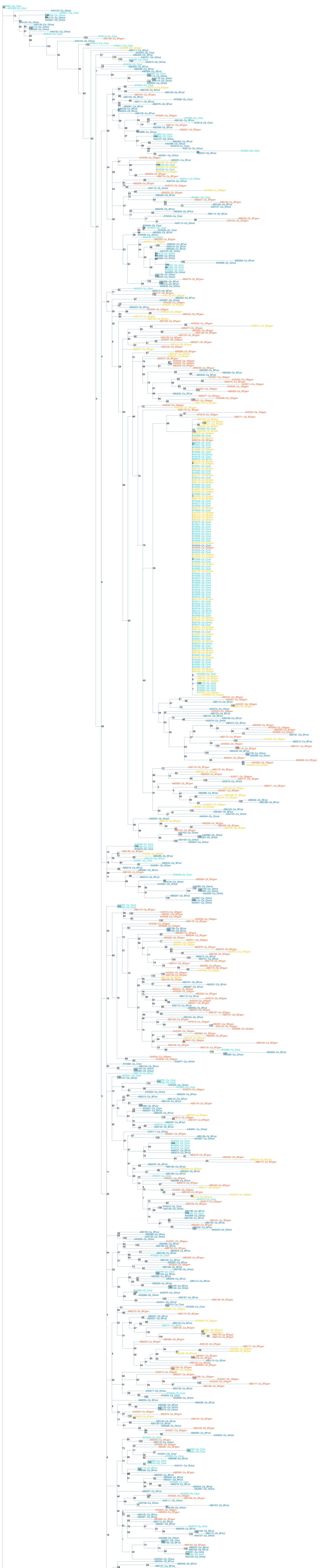

Supplementary Material SM15

C) Upstream

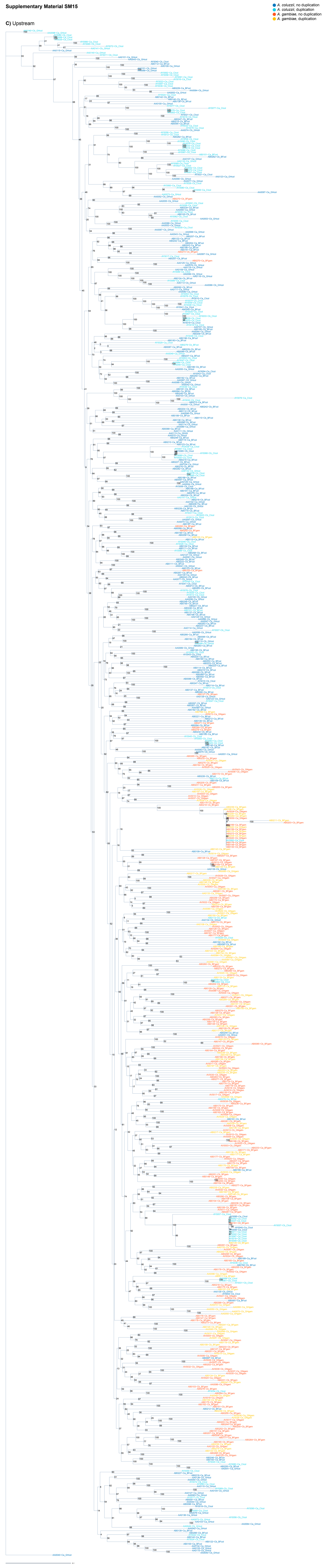

Supplementary Material SM15

D) Downstream

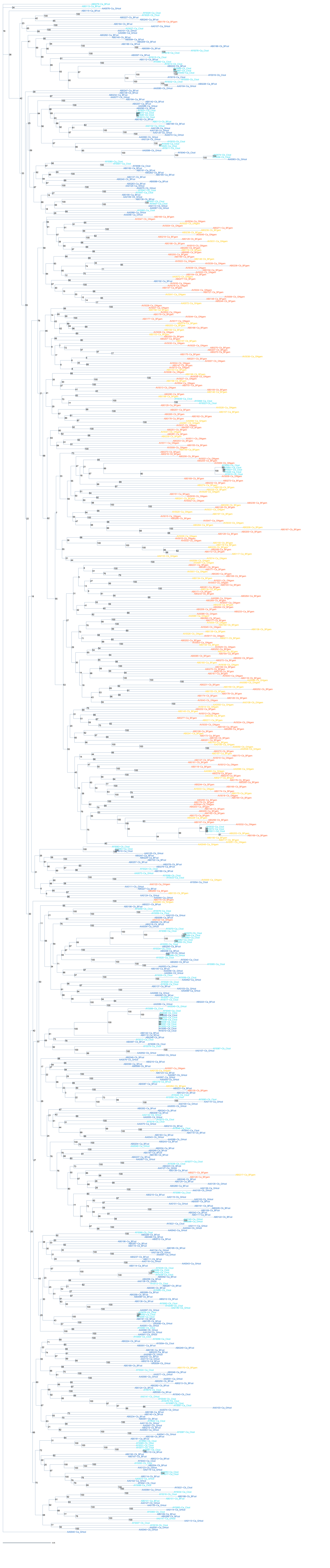

Supplement: S15 Data — Maximum-Likelihood phylogenetic analyses of haplotypes from within the Ace1 duplication (A), downstream breakpoint (B), upstream (C) and downstream (D) regions. Trees are unrooted. Tips are color-coded according to duplication presence/absence and species. UF bootstrap supports indicated in each node. (PDF) [file pgen.1009253.s015.pdf]
