# Supplementary material for: Resistance to pirimiphos-methyl in West African Anopheles is spreading via duplication and introgression of the Ace1 locus
Source: PLoS Genet. 2021 Jan 21;17(1):e1009253. doi: 10.1371/journal.pgen.1009253 (PMC7853456; doi:10.1371/journal.pgen.1009253)

Supplementary Material SM16

A) Neighbour-joining trees of haplotypes within the high-*PBS* windows

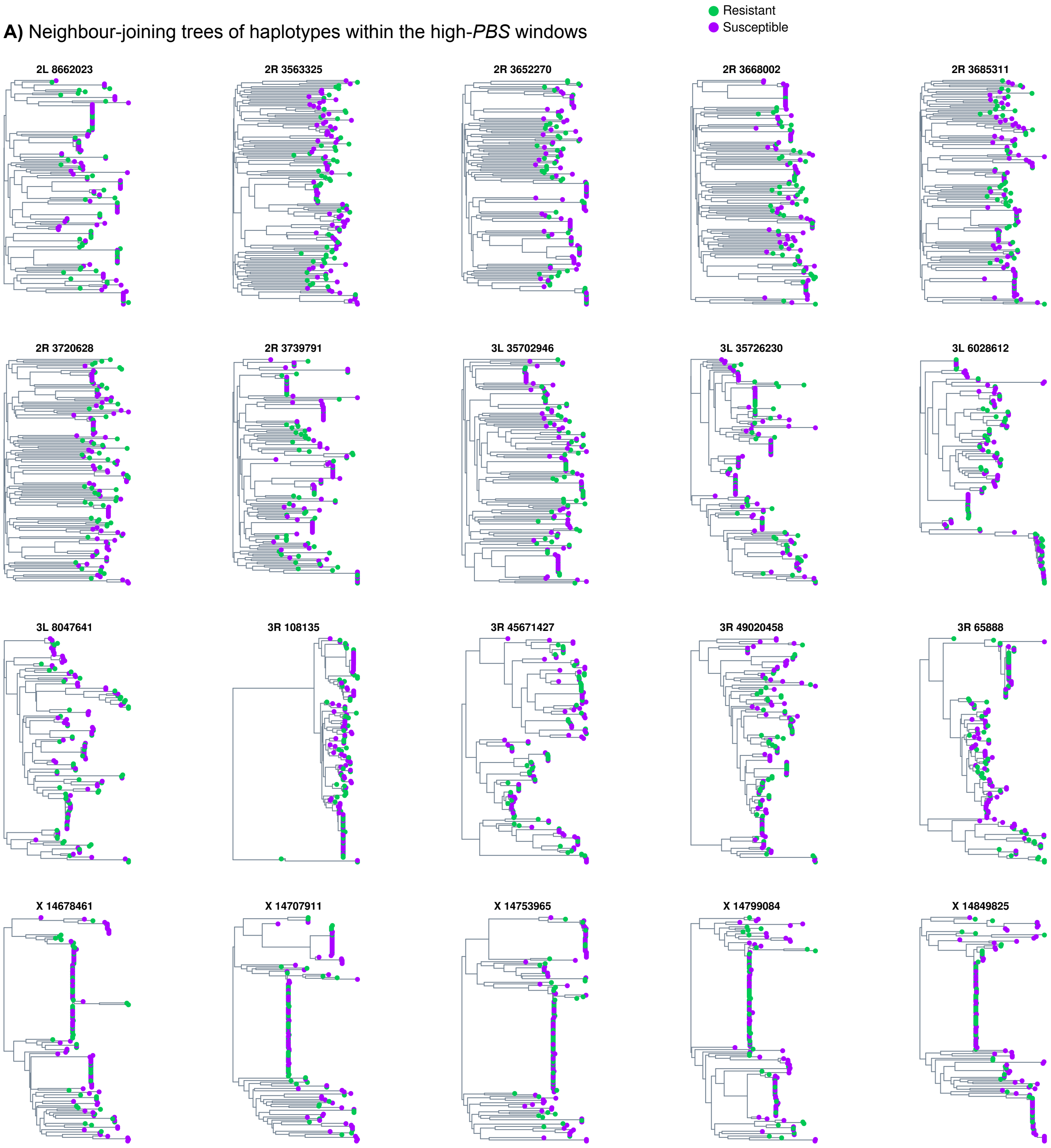

B) Profile of Garud H_{12} in the PBS peaks

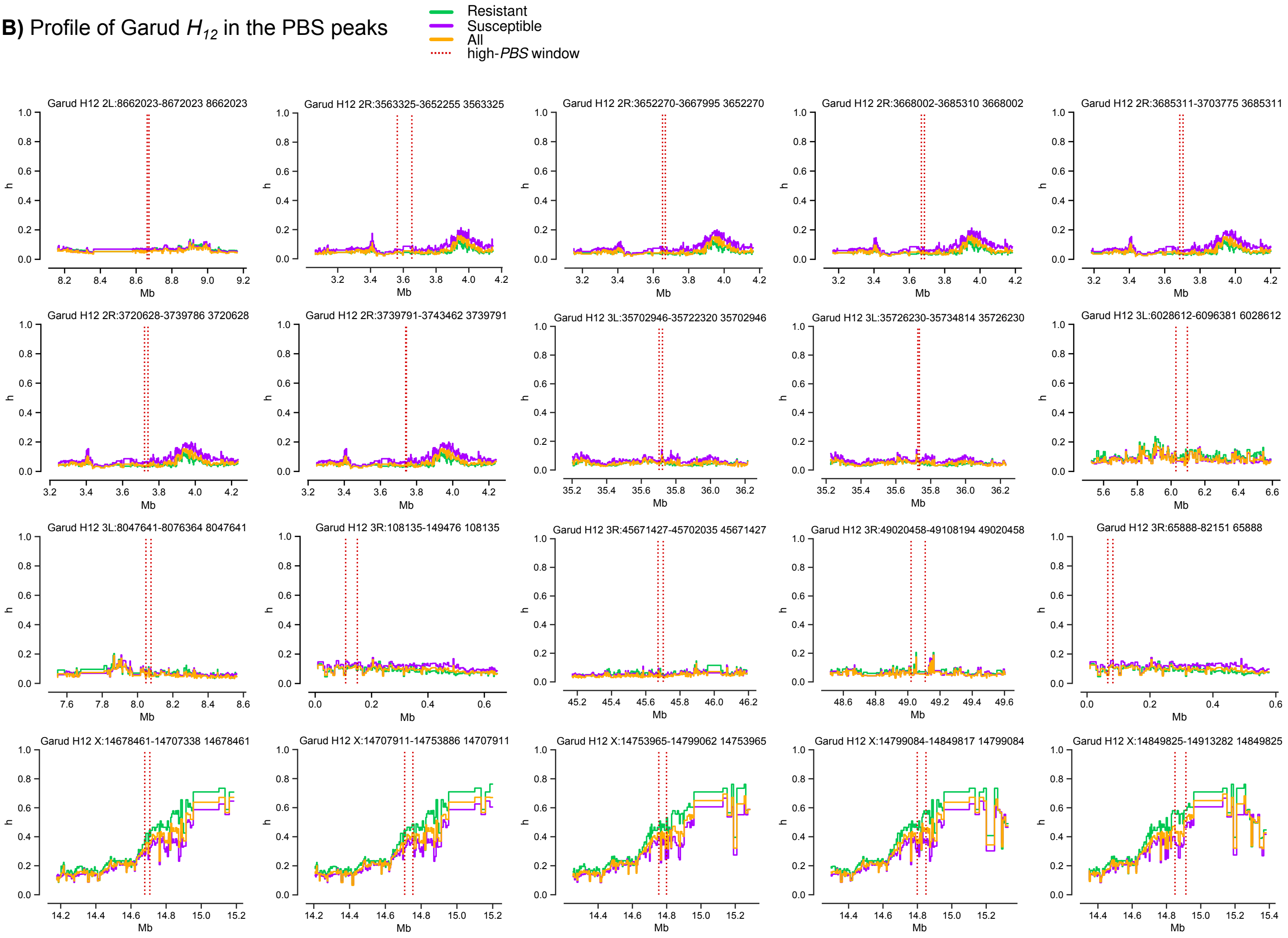

Supplement: S16 Data — A) Neighbour joining trees built from phased variants located within the PBS peaks identified between resistant and susceptible Ivorian A. coluzzii (1000 Genomes dataset). B) Profile of Garud’s H12 in these same regions. (PDF) [file pgen.1009253.s016.pdf]
