# Supplementary material for: Resistance to pirimiphos-methyl in West African Anopheles is spreading via duplication and introgression of the Ace1 locus
Source: PLoS Genet. 2021 Jan 21;17(1):e1009253. doi: 10.1371/journal.pgen.1009253 (PMC7853456; doi:10.1371/journal.pgen.1009253)

## Supplementary Material SM17

### **A)** Duplication breakpoints

| name | chrom | start | end | sequence (AgamP4, from Assogba 2016) | window (Lucas et al. 2019) |
|---|---|---|---|---|---|
| upstream_breakpoint_out | 2R | 3436893 | 3436926 | TCTTATCATGTGCTTGGTGACAATTTTGTTTTTC | 3436800 + 300bp |
| upstream_breakpoint_in | 2R | 3436927 | 3436964 | GAATCCACGGAGCGGGTGCGATTTAGGGCAGGACCGAT | 3436800 + 300bp |
| downstream_breakpoint_in | 2R | 3639803 | 3639835 | ATATTTTCCCCATTCTATTTTATCCGACGAAGC | 3639600 + 300bp |
| downstream_breakpoint_out | 2R | 3639836 | 3639873 | AAACCTGCAAAGTGGAAACAATAAGCTCTATCTGCAAC | 3639600 + 300bp |

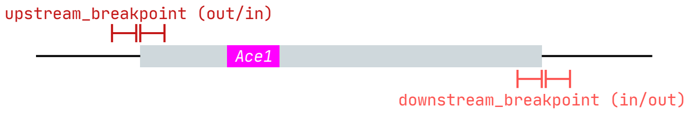


### **B)** Normalised coverage along the *Ace1* duplication

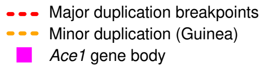


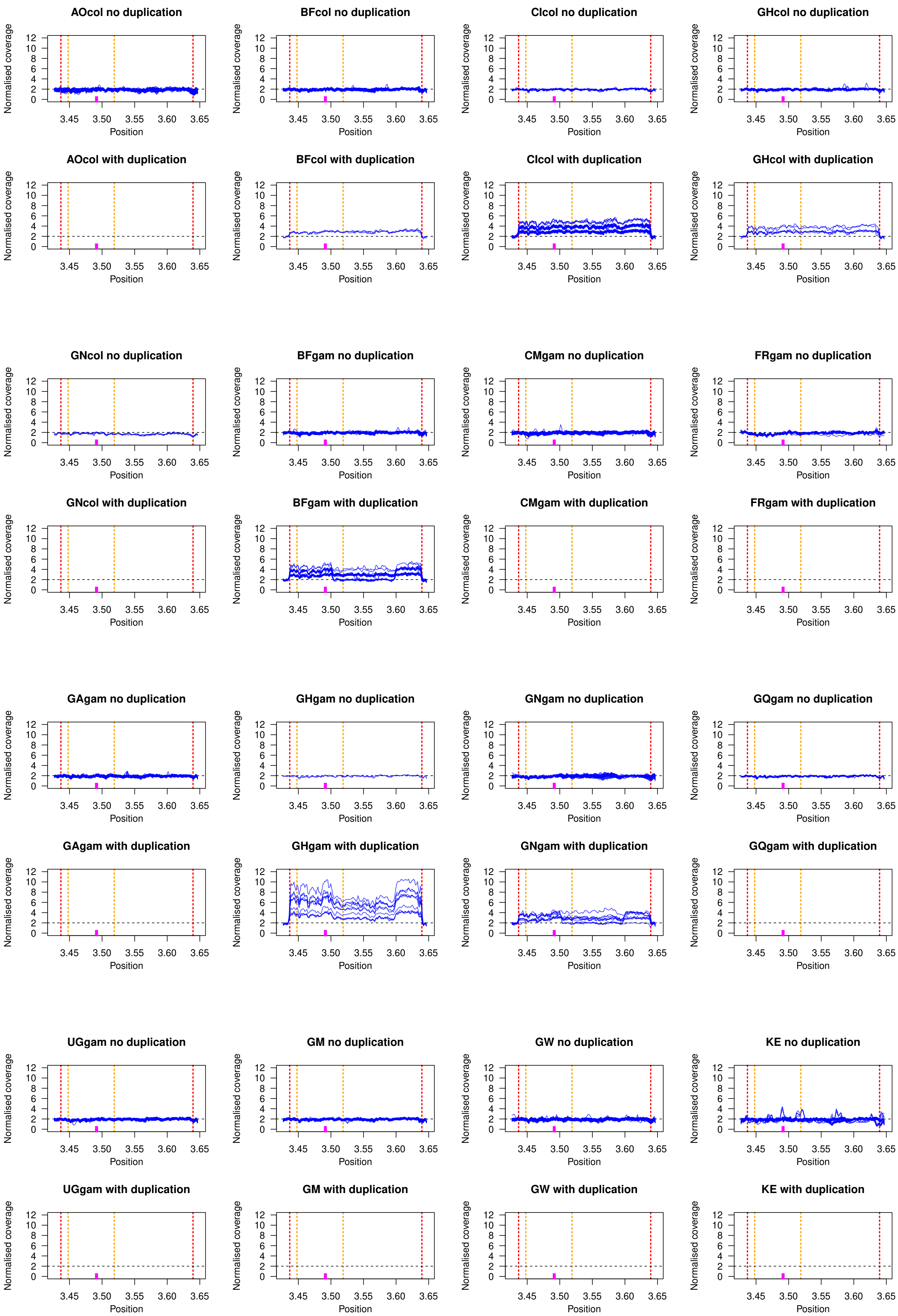

Supplement: S17 Data — A) Sequence of the duplication breakpoints described by Assogba et al. (2016) with their coordinates in the A. gambiae genome assembly (AgamP4) and the corresponding genome window in the CNV database by Lucas et al. (2019). B) Normalised sequencing coverage along the Ace1 duplication locus in each sample of the Anopheles gambiae 1000 Genomes dataset (grouped by population of origin). Detailed coverage of each sample available in S4B Data. (PDF) [file pgen.1009253.s017.pdf]
