# Supplementary material for: Resistance to pirimiphos-methyl in West African Anopheles is spreading via duplication and introgression of the Ace1 locus
Source: PLoS Genet. 2021 Jan 21;17(1):e1009253. doi: 10.1371/journal.pgen.1009253 (PMC7853456; doi:10.1371/journal.pgen.1009253)
Supplement: S18 Data — A) Distance in allelic frequencies between A. coluzzii specimens with duplications and wt A. coluzzii and A. gambiae, calculated using the three-population branch statistic in windows of 5,000 variants along the genome. Includes estimated distance (L) from within the duplication region. B) Id., using A. gambiae specimens with duplications. (PDF) [file pgen.1009253.s018.pdf]

Supplementary Material SM18

A) Distance from *A. coluzzii* duplicated specimens

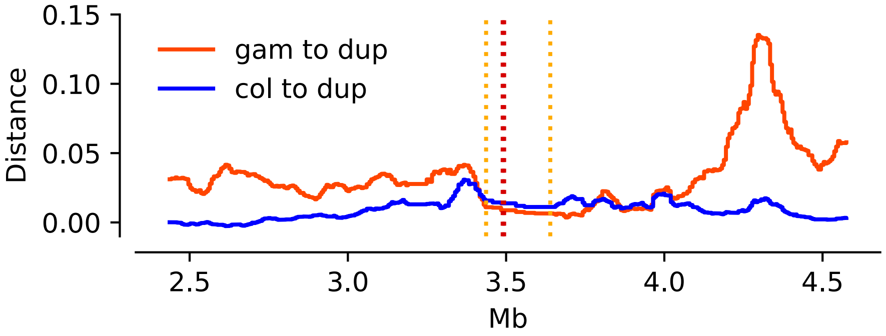

Duplication breakpoints
Ace1

Jack-knifed estimates within duplication:

L col = 0.0206925 +/- 0.0024442

L gam = 0.0134438 +/- 0.0025498

B) Distance from *A. gambiae* duplicated specimens

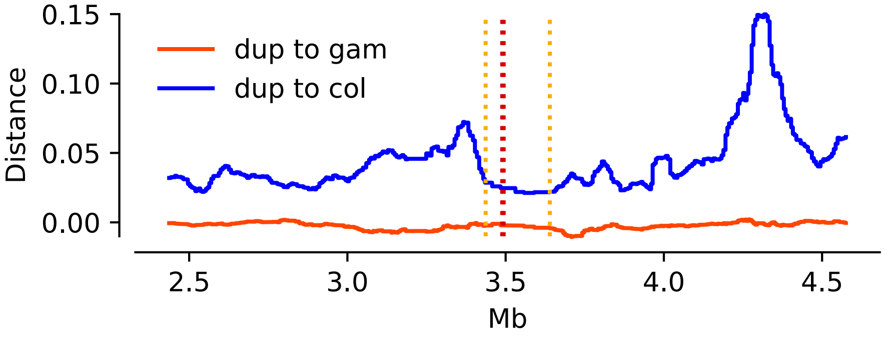

Jack-knifed estimates within duplication:

L col = 0.0332122 +/- 0.0032000

L gam = -0.0000764 +/- 0.0020300
